# Expression of TIMPs and MMPs in Ovarian Tumors, Ascites, Ascites-Derived Cells, and Cancer Cell Lines: Characteristic Modulatory Response Before and After Chemotherapy Treatment

**DOI:** 10.3389/fonc.2021.796588

**Published:** 2022-01-03

**Authors:** Ruth M. Escalona, George Kannourakis, Jock K. Findlay, Nuzhat Ahmed

**Affiliations:** ^1^ Fiona Elsey Cancer Research Institute, Ballarat, VIC, Australia; ^2^ Department of Obstetrics and Gynaecology, University of Melbourne, Melbourne, VIC, Australia; ^3^ Centre for Reproductive Health, Hudson Institute of Medical Research, Melbourne, VIC, Australia; ^4^ Department of Translational Medicine, Monash University, Melbourne, VIC, Australia; ^5^ School of Science, Psychology and Sport, Federation University Australia, Ballarat, VIC, Australia

**Keywords:** tissue inhibitors of metalloproteinases (TIMPs), metalloproteinases (MMPs), cancer stem cells (CSCs), chemo-naïve (CN), relapsed (CR), platinum resistance

## Abstract

**Background:**

The tissue inhibitors of metalloproteinase (TIMPs) and their associated metalloproteinase (MMPs) are essential regulators of tissue homeostasis and are essential for cancer progression. This study analyzed the expression of TIMP-1,-2,-3 and the associated MMPs (MMP-2,-9,-11,-14) in different Stages, Grades and World Health Organization (WHO) classifications of serous ovarian tumors, ascites, ascites-derived cells from chemo-naïve (CN) and relapsed (CR) patients, and in ovarian cancer cell lines. The status of TIMPs and associated MMPs in response to chemotherapy treatment was assessed in cancer cell lines; TCGA data was interrogated to gauge TIMPs and associated MMPs as prognostic and platinum-response indicators.

**Methods:**

The levels of TIMP-1, -2 and -3 were assessed by immunohistochemistry. The mRNA expression of TIMPs and MMPs was quantified by real time PCR (qRT-PCR). The chemosensitivity (IC_50_ values) to Cisplatin or Paclitaxel in cell lines was evaluated by MTT assay. The levels of TIMPs in ascites and cell lysates were analyzed by an ELISA assay.

**Results:**

The expression of TIMP-2 was significantly upregulated in Type 2 compared to Type 1 tumors and normal/benign ovarian tissues. TIMP-3 expression was significantly enhanced in Stage III, Grade 3 and Type 2 tumors compared to normal/benign ovarian tissues. The mRNA expression of MMP-9,-11 and -14 was significantly upregulated in Stage IV compared to normal/benign ovarian tissues. The expression of TIMP-1 was highest, followed by TIMP-2 and then TIMP-3 in CN ascites. At the cellular level, TIMP-2 mRNA expression was significantly higher in CN compared to CR epithelial cells in patients. The expression of TIMP-1 and -2, MMPs and cancer stem cells (CSCs) were upregulated in response to chemotherapy treatments in cancer cell lines. Interrogation of the TCGA dataset suggests shifts in platinum responses in patients consistent with genetic alterations in TIMP-2, -3 and MMP-2, -11 genes in tumors; and decreased overall survival (OS) and progression-free survival (PFS) in patients with altered MMP-14 genes.

**Conclusions:**

TIMPs and related MMPs are differentially expressed in serous ovarian tumors, ascites, ascites-derived cells and ovarian cancer cell lines. Chemotherapy treatment modulates expression of TIMPs and MMPs in association with increased expression of genes related to cancer stem cells.

## 1 Introduction

Epithelial ovarian cancer development and progression is a multi-stage process that originates in the epithelial cells of the female reproductive tract and in the majority of cases follows a transcoelomic route involving mainly organs and tissues within the peritoneal cavity (1, 2). Metastasis in ovarian cancer starts with cancer cells that originate either on the surface epithelium of the ovaries or the Fallopian tubes and metastasize to the peritoneal cavity by attaching on the mesothelial layer with successive invasion on the extracellular matrix that underlies the stroma (1, 3). In that scenario, the compression and sheer pressure of ascites in 30-40% patients plays an essential role in the spread of cancer (4–7). Hence, understanding the mechanisms by which epithelial ovarian tumors, the associated ascites, and its cellular components evolve and induce changes in the key regulatory molecules to promote cancer progression is essential to design targeted treatment.

Ovarian tumors are classified in three main ways. Federation of Gyneacology and Obstetrics (FIGO) Stages is based on the surgical evaluation of tumors and is an important predictor of cancer dissemination and long-term survival in patients (8). However, the histopathological classification, commonly known as the Shimizu/Silverberg system, based on the architecture of tissues (glandular, papillary, or solid), degree of nuclear atypia, and mitotic index, is essential for the evaluation of appropriate treatments (9, 10). Lately, much emphasis has been placed in understanding the source of epithelial ovarian cancer, resulting in the introduction of WHO classification, based on the origin, the differences in histologic subtypes and the molecular/genetic alterations in tumors (11–14). According to WHO classification, Type 1 tumors (low-grade), derived mainly from the surface epithelium, harbor mutations in KRAS, BRAF or HER2/neu genes are slow growing tumors, and include endometriod, clear cell, mucinous and low-grade serous subtypes (15). In contrast, Type 2 tumors (high-grade) evolve from the intra-epithelium of the Fallopian tube, are proliferative and aggressive, carry a p53 mutation, HER2 gene amplification and AKT2 overexpression, and constitute mostly the high-grade serous subtype (16).

Almost 80% of ovarian cancer patients present with an advanced-stage disease (3). Primary cytoreduction surgery, followed by chemotherapy is a standard treatment, which in most cases gives patients a short reprieve (few months), but they relapse within few months. The current standard of chemotherapy treatment is usually platinum (cisplatin or carboplatin). However, different combinations of other chemotherapy reagents are given such as paclitaxel, cyclophosphamide, doxorubicin and gemcitabine, which are the most common second or third line of treatments (17). Most ovarian cancer patients are initially responsive to chemotherapy but become increasingly resistant to treatment with each episode of recurrence and eventually become totally chemoresistant and untreatable (18). Hence, to achieve a much-needed outcome in patients’ therapy, a study of the factors in the ovarian tumor microenvironment that includes an investigation of the therapy resistant tumorigenic cells residing in primary tumors or in the ascites is essential. Of particular interest are members of the metzincin family, MMPs and TIMPs, crucial for tissue remodeling in response to diseased conditions, including cancer (19).

MMPs are a family of proteinases that play a major role in remodeling of the extracellular matrix (ECM) as breakdown and rapid turnover of ECM molecules is crucial for cancer progression and metastasis (20–22). Human somatic cells express 23 different MMPs, which are essential for tissue development and homeostasis. In disease progression, including cancer, certain MMPs are deregulated to meet the homeostatic demand of the diseased tissue, which requires excessive proliferation, migration and infiltration of diseased cells to different tissues requiring ECM remodeling (23, 24). As MMPs regulate the ECM protein turnover, necessary for cancer progression their activities in most cases are strictly regulated by their endogenous inhibitors, such as TIMPs.

A family of four endogenous TIMPs regulate the protease activities of MMPs in the tumor microenvironment. Each TIMP binds multiple MMPs in 1:1 stoichiometric ratio and inhibits their activities. However, recent studies have shown MMP-independent functions for TIMPs (25). In that regard, TIMPs have been shown to regulate a whole range of signaling pathways including mitogen activated protein kinase (MAPK), cyclic adenosine monophosphate (cAMP)-protein kinase A and activation of Ras pathways to promote cell growth (25–27).

Recent cancer studies suggest an imbalance in the expression of MMP: TIMP ratio to be an indicator of progression of disease. For example, an imbalance in the expression of MMP-9 and TIMP-1 was noted in gastric and laryngeal squamous cell carcinomas (28, 29), while an imbalance in expression of MMP-2 and TIMP-2 has been reported for hepatocellular carcinoma (30). In colorectal cancer, disproportionate expression of MMP-8 and TIMP-1 has been reported (31). In ovarian cancer, high mRNA expression of MMP-2, -9, -14 and TIMP-2 was reported in ovarian tumors, and correlated with low survival in patients, indicating that the expression of these genes may act as suitable nominators of survival in advanced-stage patients (32). Abdominal ascites in ovarian cancer patients is enriched in MMP-2, -9, and -14 which play a major role in peritoneal dissemination (33). In addition, expression of MMP-9 or -14 in epithelial tumor cells of ascites have been linked with poor survival in patients (33, 34). Although co-expression of TIMPs and associated MMPs have been reported in ovarian tumors and ascites (35, 36), their correlation with disease progression and their role in chemoresistance is still ambiguous. Hence, the aim of this study was to extensively analyze the expression patterns of TIMPs and associated MMPs in ovarian tumors, ascites, ascites-derived epithelial and mesenchymal cells from CN and CR patients and in ovarian cancer cell lines, before and after chemotherapy treatments. Assessment of a TCGA dataset was performed to test the involvement of TIMPs and MMPs as prognostic and platinum response indicators. Our data supports the involvement of TIMPs and MMPs in ovarian cancer progression and chemoresistance.

## 2 Materials and Methods

### 2.1 Collection of Tumors and Ascites From Patients

#### 2.1.1 Ethics Approval

This project (Project 09/09) was approved by the Research and Ethics Committee of Royal Women’s Hospital (RWH), Melbourne, Australia. All participating patients signed patient consent forms.

#### 2.1.2 Collection of Ovarian Tumors

Epithelial serous ovarian tumors were collected from patients admitted for surgery after diagnosis of ovarian cancer at The Royal Women’s Hospital (RWH), Melbourne, Australia. Benign tumors or normal ovaries were collected from patients undergoing abdominal hysterectomy or bilateral salpingo-oophorectomy due to pre-existing medical conditions. All tissues were fixed in 4% paraformaldehyde or snap frozen at the time of collection. Tumor samples were snap frozen and stored at -80°C to be used later for RNA extraction. Information on patients such as tumor Grade, Stage and Type of tumor were obtained from RWH pathology reports. Patients participating in this study were chemo-naïve and did not undergo any form of therapy. Clinical information on patients used for the immunohistochemistry and mRNA studies are described in **Supplementary Tables 1A, B**).

#### 2.1.3 Collection of Ascites

Ascites samples were obtained from patients diagnosed with high-grade serous ovarian cancer undergoing treatment at either RWH, Melbourne, Australia or at Monash Medical Centre, Clayton, Australia. Chemo-naïve ascites (CN) were collected from patients at initial cancer diagnosis. Recurrent ascites (CR) were collected from patients at the time of disease recurrence following chemotherapy treatment. Clinical information on each ascites used in the study is described in **Supplementary Table 2**.

### 2.2 Cell Culture

#### 2.2.1 Preparation of Tumor Cells From the Ascites of Ovarian Cancer Patients

Ascites samples were processed within 24 hours of collection. Cells in the ascites were separated by the method developed in our laboratory as described previously (37). Briefly, approximately 100-500mL of ascites was centrifuged and the supernatant collected and stored at -80°C. Red blood cells from the cell pellet were removed by hypotonic shock. The remaining cells were suspended in a 1:1 ratio of MCDB131 (Life Technologies, CA, USA) and DMEM (Sigma-Aldrich), supplemented with 10% (v/v) heat inactivated FBS (Thermo Fisher Scientific, MA, USA), 2mM L-glutamine (Invitrogen Corporation, CA, USA), and an antibiotic combination of 1% (v/v) streptomycin and penicillin (Invitrogen Technologies, Vic, Australia). Approximately 1x10^5^-1x10^6^ tumor cells per well were seeded onto 6-well Corning^®^ Ultra-Low attachment plates (Sigma-Aldrich, St. Louis, MO, USA) and incubated at 37°C in 5% CO_2_. Seeded cells grew into two distinct populations: the non-adherent epithelial cells and adherent mesenchymal population were collected and frozen in TRIzole (ThermoFisher Scientific, Melbourne, Australia) for RNA extraction.

#### 2.2.2 Ovarian Cancer Cell Lines

Thirteen established human ovarian cancer cell lines: AOCS1, CAOV3, COV318, HEY, JHOS2, JHOS4, OVCA433, OVCA429, OVCAR4, OVCAR5, OVKATE, SKOV3, TOV21G and a cell line derived from a normal Fallopian epithelium, FT282, were a kind gift from Prof. David Bowtell (Peter MacCallum Cancer Centre, Parkville, Australia). Cell lines were tested for mycoplasma contamination by the Victorian Infectious Disease Reference Laboratory (Parkville, Australia).

Cell lines were sustained in either RPMI-1640 (Sigma-Aldrich, Sydney, Australia), or DMEM, or 1:1 ratio of medium MCDB131 and DMEM, or 1:1 ratio of medium F-12 and DMEM. Cell line growth media were supplemented with 1% (v/v) 2mM L-glutamine, an antibiotic-antimycotic combination of 1% (v/v) Fungizone, Streptomycin and Penicillin and 10% (v/v) FBS (Thermo Electron, Melbourne Australia) with the exception of the FT282 cell line which was supplemented with Ultroser™ G serum substitute USG (PALL, Life Sciences, NY, USA) instead of FBS. For cell lines, JHOS-2 and JHOS-4, the culture media was also supplemented with 1% (v/v) non- essential amino acids (NEAA). Cells were cultivated as adherent cultures on sterile Corning^®^ CellBIND^®^ 25cm^2^, 75cm² or 175 cm² cell culture flasks (Life Sciences, Australia) at 37°C in 5% CO_2_ humidity.

### 2.3 Immunohistochemical Analyses of TIMP-1, -2 and -3 in Ovarian Tumors

Ovarian tumors were outsourced to the Anatomical Pathology Laboratory Services, The Royal Children’s Hospital, Melbourne, Australia. Paraffin embedded tissues were sectioned at 4μm thickness and stained with either TIMP-1, -2 or -3 polyclonal antibodies (1:100, Abcam, Cambridge, UK and Dako, Santa Clara, USA) and an OptiView DAB IHC detection kit (Ventana Medical Systems, Inc, Arizona, USA). The samples were processed on a Ventana Benchmark Immunostainer (Ventana Medical Systems, Inc, Arizona, USA) as described previously (38). Negative controls were prepared by incubating samples in primary antibody diluents followed by the secondary antibodies. Sections of human placental and tonsil tissues were used on each slide as positive controls. Sections were assessed microscopically for positive DAB staining in conjunction with positive CA-125 staining. Once areas of interest were identified, approximately eight random areas were drawn using the software and then measured for total DAB positivity staining as part of the software analysis. Total DAB positivity was divided by the area (µm^2^) to obtain positivity over area for each reading, and average staining for each block was calculated. This procedure was repeated for the negative controls and the results subtracted from the average DAB positivity over area of the antibody of interest. Stained slides were scanned using the Aperio Scanscope XT (Aperio-Leica Microsystems Pty Ltd) and imaged using the Aperio ImageScope v12.3.2.8013 software (Leica Biosystems Pathology Imaging 2003-2016). Results were then plotted on a bar graph using PRISM software, analysed, and sorted according to FIGO Stages, Silverberg grades and WHO classification.

### 2.4 Methyl Thiazol Tetrazolium (MTT) Assay

MTT assay is a colorimetric assay used to quantify the survival of viable cells (38, 39). In brief, 3 x 10^4^ cells were seeded into 96-well plates overnight. Cell lines were treated with either paclitaxel (PTX) (EbeweR, SANDOZ, Novartis, Basel Switzerland) or cisplatin (CIS) (Accord Healthcare Pty Ltd, Melbourne, Australia) and MTT assay was performed by replacing culture media with 100µL of thiazolyl blue tetrazolium (MTT) solution (Sigma-Aldrich) dissolved in 1x PBS solution (0.5mg/mL final concentration) (Sigma-Aldrich). Cells were incubated for 2 hours at 37°C in 5% CO_2_ humidity. Media was discarded and replaced with 100µL of dimethyl sulfoxide (DMSO). Absorbance was read at OD595nm using the CLARIOstar Plate Reader (BMG Labtech, Germany) and data was analysed by MARS Data Analysis Computer Software (BMG Labtech, Mornington, Victoria, Australia).

### 2.5 Human TIMP ELISA for Measurement of TIMP-1, -2 and -3 in Ascites and Cell Lysates

ELISA (enzyme-linked immunosorbent assay) was performed using the MILLIPLEX^®^ MAP Human TIMP Magnetic Bead Panel 2, as per manufacturer’s instructions (EMD Millipore Corporation, USA). Briefly, 25µl of diluted ascites (1:80) or 40μg of cell lysates were added to individual wells of 96-well plates in duplicate. After treatment with Wash Buffer, detection antibodies were added for 1 hour. This was followed by Streptavidin-phycoerythrin treatment for another 30 minutes, two washing steps and treatment with 100µl of Bio-Plex^®^ Sheath fluid (Bio-Rad, Austin, Texas, USA) and run on a Luminex 200TM (Bio-Rad, Texas, USA) equipped with BioPlex Manager 5.0 software (Bio-Rad,Texas, USA).

### 2.6 RNA Extraction, Quantitative and Relative Real-Time PCR (qRT-PCR)

RNA was extracted from snap frozen ovarian tumor sections, ascites-derived epithelial and mesenchymal cells and ovarian cancer cell lines stored in TRIzol^®^ reagent (Ambion-Life Technologies, CA, USA) by the chloroform: phenol method as described previously (40). Five hundred ng of total RNA was reverse transcribed using the high-capacity cDNA Reverse Transcription Kit (Applied Biosystems, CA, USA), and both relative and absolute qRT-PCR amplification was performed using the Applied Biosystems ViiA 7 Real-Time PCR (Thermo Fisher Scientific, NSW, Australia) as described previously (40–42). Briefly, for absolute qRT-PCR method, samples were analysed against a known absolute quantity of the gene (fg), and these results were then standardized against an absolute quantity of 18S (fg) and this method was used for comparing tissue samples of different origin. While relative PCR compared Ct values relative to 18S Ct values (δΔCt) and was only used for samples within the same origin. All PCR reactions were performed in triplicate. The sequences and accession numbers of genes analyzed and primers used are listed in **Supplementary Table 3**. Data are presented as absolute values (fg) normalized to 18S or relative expression normalized to the housekeeping gene 18S.

### 2.7 Statistical Analysis

Data are presented as mean ± standard error (SEM). An unpaired Mann-Whitney’s non-parametric t-test was used when comparisons were made between two groups. A One-Way ANOVA (Tukey’s or Dunnett’s multiple comparison tests) was used when more than two treatment groups were compared. xCELLigence data was analyzed by linear regression analysis, and presented as the standard deviation (SD) of the mean. For statistical significance, probability levels of p<0.05(*), p<0.01(**), p<0.001 (***) and p<0.0001 (****) were used. Data was analyzed by using Graph Pad PRISM software and Microsoft Excel 2016. All experiments were performed at least three times (unless otherwise indicated) in triplicate.

## 3 Results

### 3.1 Expression of TIMPs in Serous Ovarian Carcinomas Using TCGA Platform

**Figure 1:**
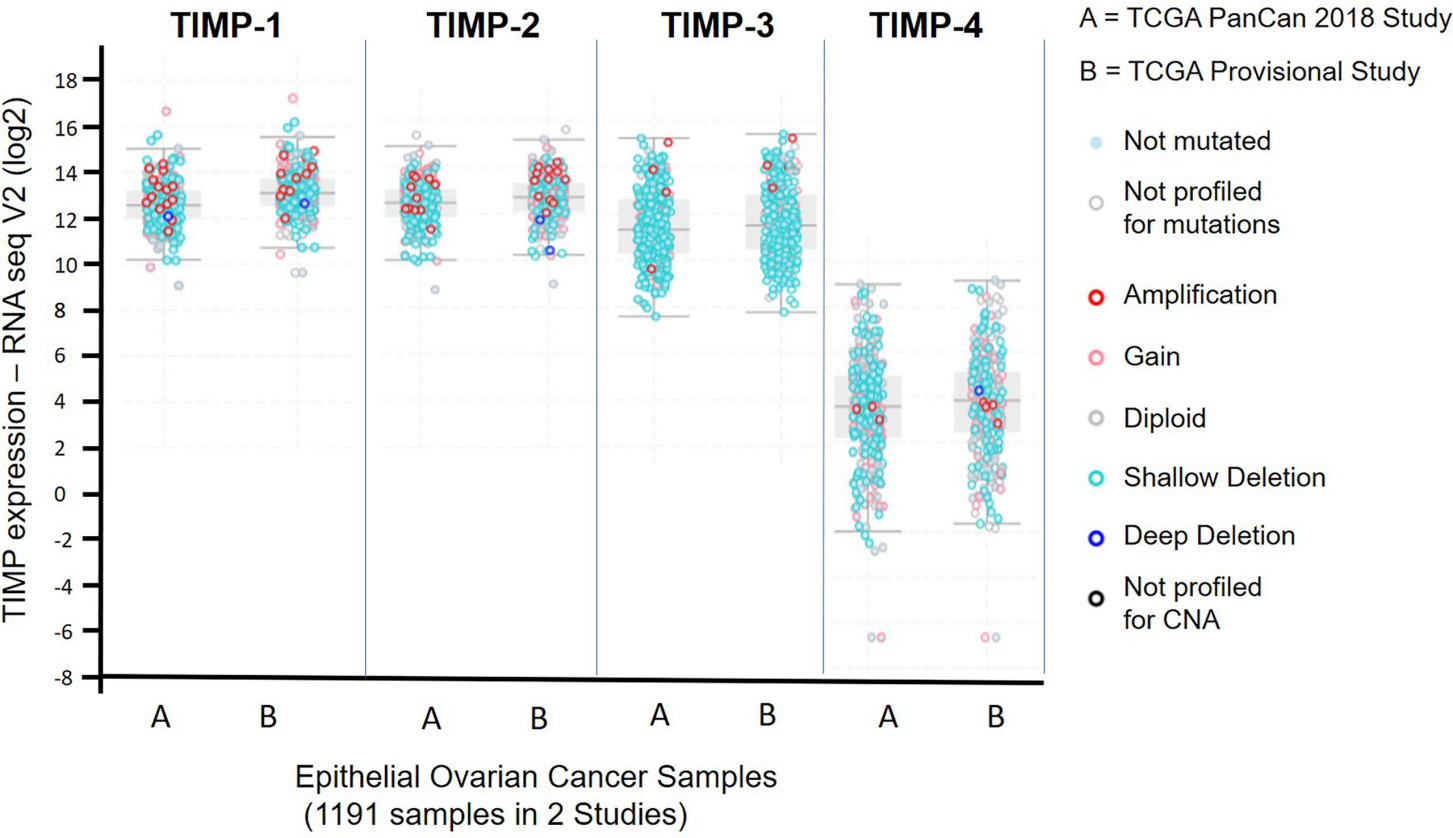
mRNA expression of TIMP-1, -2 and -3 in 1191 epithelial ovarian serous cystadenocarcinoma patients from two different studies published in the TCGA database: (A) the TCGA PanCan 2018 study (585 patient samples); and (B) the TCGA Provisional study (606 patient samples). Light blue filled dots, indicate genes not mutated, open light grey dots specify samples not profiled for mutation, open dark red dots show a gene amplification, open pink dots signify a mutation gain, open grey dots a diploid gene, light open blue dots a shallow deletion, a dark open circle indicates a deep deletion, and a dark black open circle specifies samples not profiled for copy number alteration (CNA).

The Cancer Genome Atlas (TCGA) is a revolutionary public cancer genomic platform (http://www.cbioportal.org) that contains genomic information on hundreds of molecularly characterized tumor samples (43, 44). This genomic platform was used to assess the abundance of the TIMP genes in untreated serous ovarian cancer samples. **Figure 1** demonstrates the data generated from 1191 ovarian serous cystadenocarcinoma patients from two different studies; (A) the TCGA PanCan 2018 study (585 patient samples) and (B) the TCGA Provisional study (606 patient samples). Overall, there were no difference in gene expression between A and B, indicating consistency in the data sets from two different studies. The data set indicates that TIMP-4 mRNA was expressed in lesser amounts than TIMPs 1-3 in ovarian carcinomas. The highest mRNA expression was observed in TIMP-1 [median ~13.11 RNA-Seq V2 (log2)], followed by TIMP-2 [median ~12.71 RNA-Seq V2 (log2)] and TIMP-3 [median ~11.51 RNA-Seq V2 (log2)]. This reiterated the fact that TIMP-4 has a lesser ovarian tissue distribution, and as reported previously is expressed more in heart, brain and testes tissues (45, 46). Secondly, there is a higher incidence of alterations (gene amplification and gene mutation) in TIMP-1 and TIMP-2 genes while TIMP-3 and TIMP-4 genes offered less proportion of gene alterations in the patient cohorts (**Figure 1**). Based on these findings we focused on quantifying the protein and mRNA expression of TIMP-1, -2 and -3 in the untreated primary serous ovarian tumor samples.

### 3.2 Expression of TIMPs in Serous Ovarian Carcinomas

**Figure 2:**
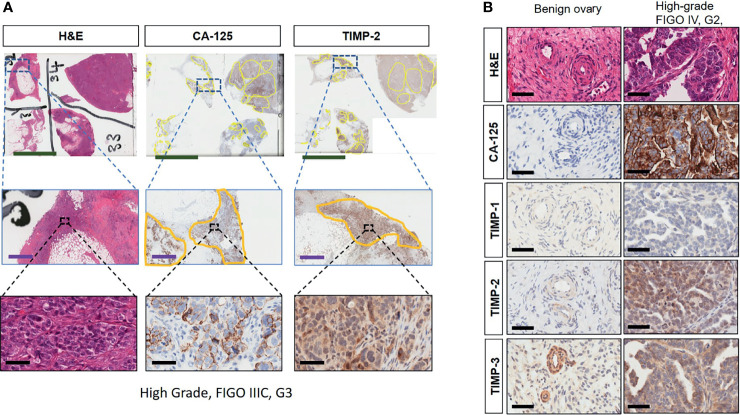
**(A)** Expression and co-localization of TIMP-2 and CA-125 in high-grade serous ovarian tumors compared to benign serous tumors. All images are representative of the same high-grade, Stage III and Grade 3 serous ovarian tumor. Column 1 is representative of that tumor stained with H&E. Columns 2 and 3 represent corresponding CA-125 and TIMP-2 staining on the same area of that tumor. Each row indicates different magnification, first row indicates magnification 0.2X, scale green bar = 9mm; second row a magnification 2X, scale purple bar = 1mm; third row magnification 40X, scale black bar = 50µm. **(B)** Matching sections showing H & E stain, CA-125, TIMP-1, -2 and -3 staining of benign and high-grade tumor. Magnification 40X, scale black bar = 50µm.

**Figure 3:**
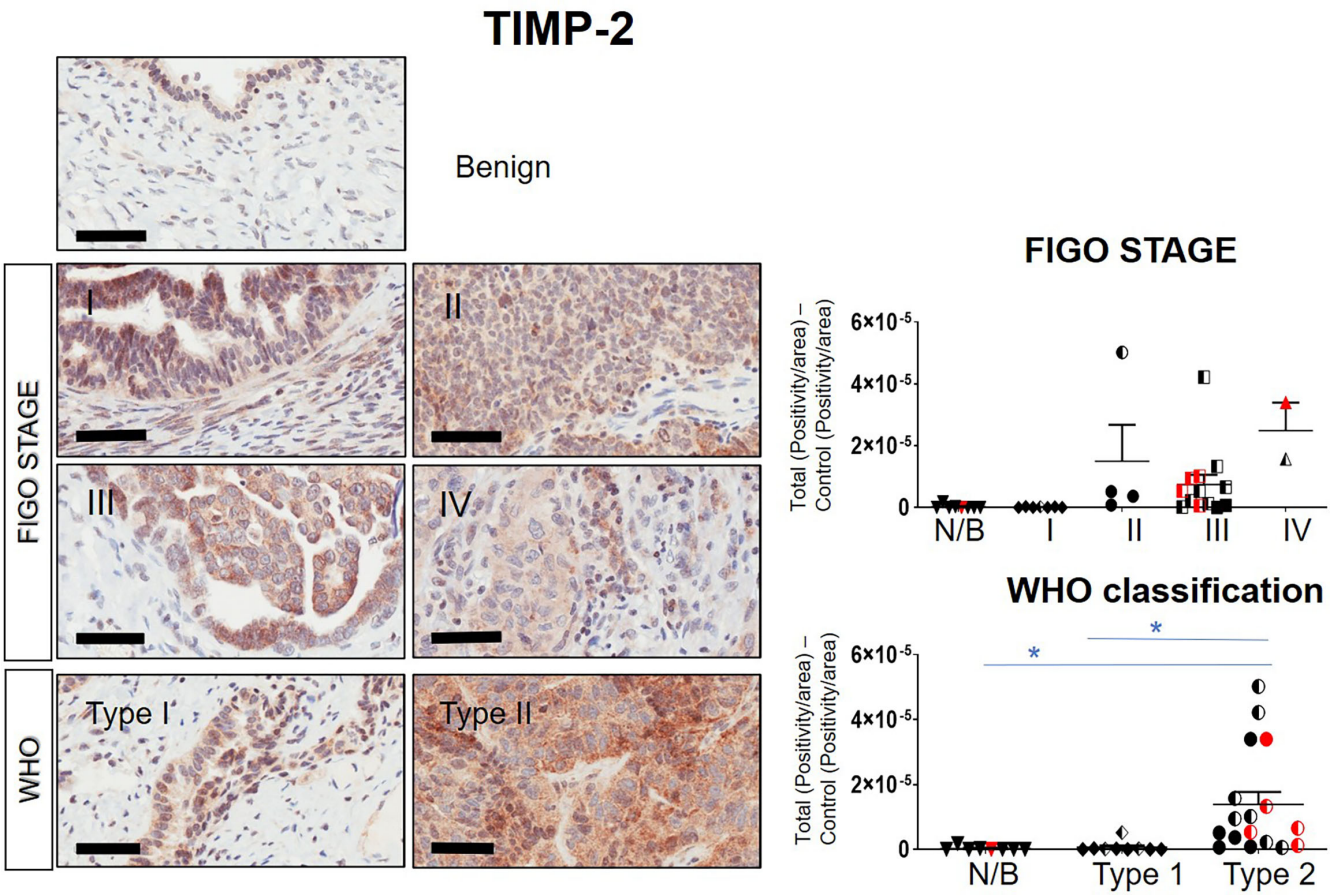
Expression of TIMP-2 in primary ovarian tumors according to different tumor classifications. Representative images of TIMP-2 staining in primary ovarian tumors (n=26) and benign ovarian tumors (n=8). Images of samples are classified into Stages and WHO classification. Magnification 40X, scale-bar=50µm. Graphs represent DAB positivity over area divided by the negative control for each patient tissue block. Red points indicate BRCA positive patients and black/white points indicated patients with ascites present at the time collection. Significance was determined by One-way ANOVA (Tukey’s multiple comparison test) and indicated by *p <0.05.

**Figure 4:**
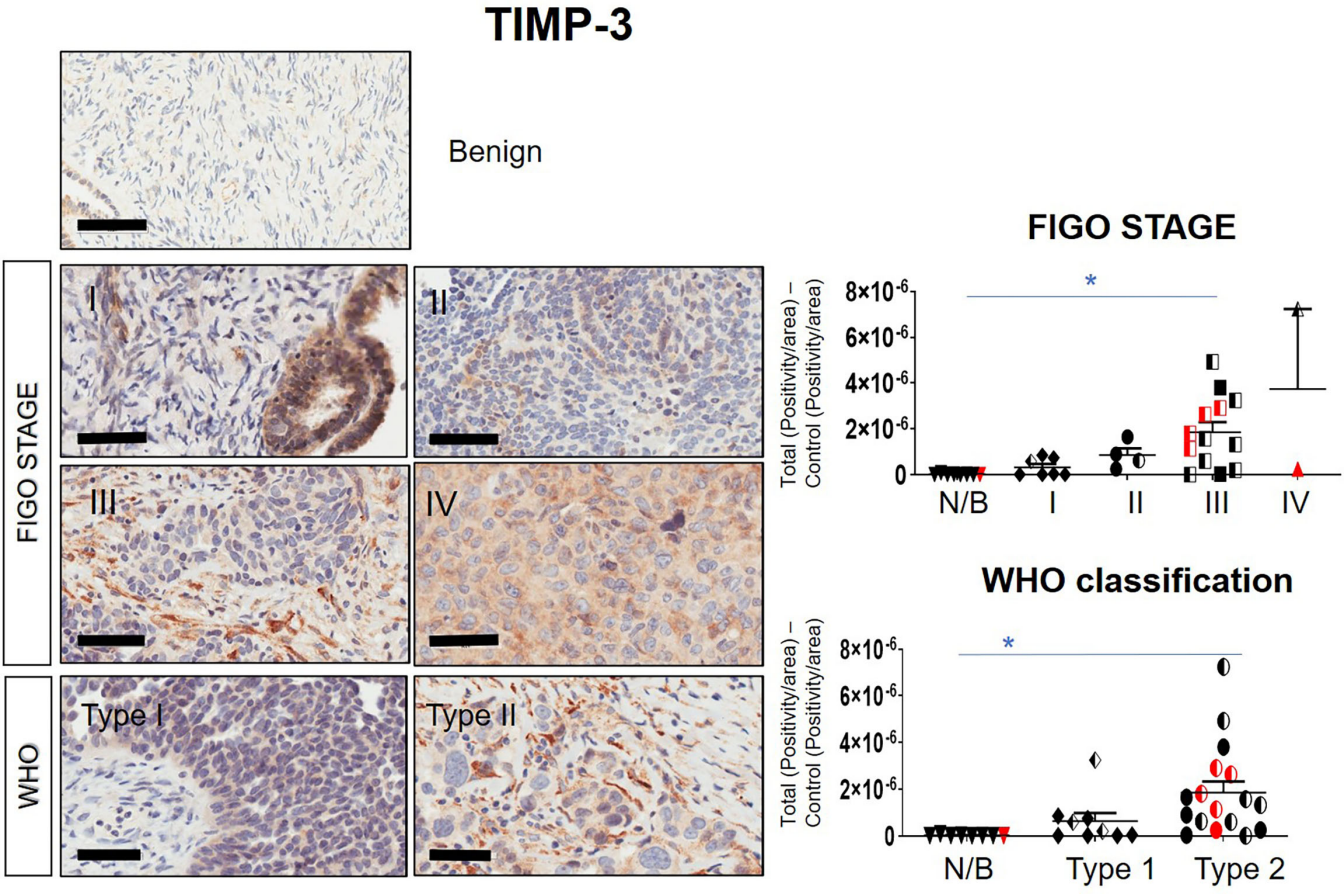
Expression of TIMP-3 in primary ovarian tumors according to different tumor classifications. Representative images of TIMP-3 staining in primary ovarian tumors (n=26) and benign ovarian samples (n=8). Images of samples were sorted into Stages and WHO classification. Magnification 40X, scale bar = 50µm. Graphs represent DAB positivity over area divided by the negative control for each patient tissue block. Red points indicate BRCA positive patients and black/white points indicated patients with ascites present at the time collection. Significance between normal/benign tissues and carcinomas was determined by One-way ANOVA (Tukey’s multiple comparison test) and is indicated by *p <0.05.

Immunohistochemistry staining of TIMP-1, -2 and -3 was performed on 36 benign and ovarian tumor samples. In almost all tumors TIMP-1, -2 and -3 expression was noted in the epithelial tumor cells also co-expressing CA-125 (**Figure 2**). An example of how the sections were analyzed is provided in **Figure 2A** for TIMP-2, while **Figure 2B** provides representation of the TIMPs -1, -2, -3 at a higher magnification coinciding at the same region as CA-125. The differences in the expression of TIMP-1, -2 and -3 were analyzed according to Stages, Grades and WHO classifications. TIMP-1 expression was very low in ovarian tumors and was not significantly different under any of the above categories (**Supplementary Figure 1**). TIMP-2 expression, on the other hand, showed significant differences using the WHO classification. Significant differences in the expression of TIMP-2 were observed between normal/benign and Type 2 ovarian tumors (**Figure 3**). However, no significant difference in TIMP-2 expression between different Grades or Stages (**Figure 3**, data not shown for grades) was observed. In contrast, significant upregulation of TIMP-3 expression in Stage III, Grade 3 and Type 2 tumors, compared to normal/benign tissues was observed (**Figure 4**, data not shown for grades). An upregulation of TIMP-3 in FIGO Stage IV compared to normal/benign tissues was also noted in two available samples (**Figure 4**).

BRCA1/2 mutations were observed in patients diagnosed with either Stages III/IV or Type 2 tumors (red dots in **Figures 2**, **3** and **Supplementary Figures 1**). The presence of ascites was noted in patients categorized under both Type 1 and 2 as well as Stages II/III tumors (black and white dots in **Figures 2**, **3** and **Supplementary Figure 1**).

Overall, the levels of TIMP-3 increased with Stage, WHO Type and Grade compared to control tissues. TIMP-2 only increased in the WHO Type 2, while no change was noted in TIMP-1 across all classifications.

### 3.3 mRNA Expression of TIMPs and Associated MMPs in Primary Serous Ovarian Carcinomas Classified by Stages, Grades or WHO Types

**Figure 5:**
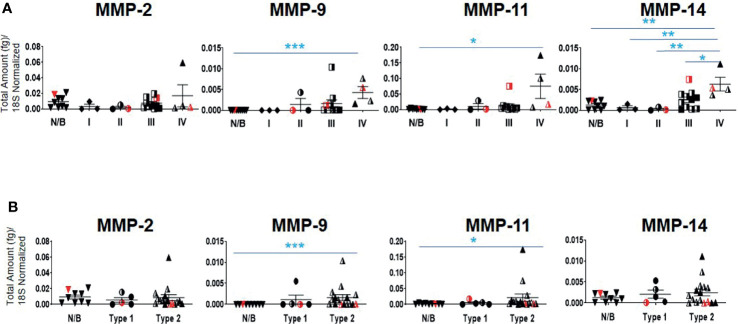
mRNA expression of MMP genes in primary ovarian tumors. Graphs represents MMP-2, -9, -11 and -14 mRNA expression across **(A)** Stages I-IV, and **(B)** WHO Types 1 and 2 classifications. The total mRNA expression (fg) was normalized to 18S in 21 ovarian serous epithelial and 9 benign ovarian tumors (N/B). Red points indicate BRCA positive patients and black/white points indicated patients with ascites present at the time collection. Values are mean ± SEM; significance was determined by One-way ANOVA (Tukey’s multiple comparison test) and is indicated by *p<0.05, **p<0.01, ***p<0.001.

Twenty-one serous ovarian tumors and nine benign/normal ovarian tissues were included in this study. There were no significant differences between the mRNA expression of TIMP-1, -2 and -3 between the Stages, Grades and WHO Types (**Supplementary Figure 2A**). The mRNA expression of MMP-2, -9, -11 and -14 was also studied, as these are the major MMPs regulated by TIMP-1, -2 and -3 (45, 47). The mRNA expression of MMP-9,-11 and -14 was significantly upregulated in Stage IV tumors compared to normal/benign tissues (**Figure 5A**); while no significant change for MMP-2 mRNA expression was noted (**Figure 5A**). No significant change was also observed according to the Grading for any of the MMPs tested (**Supplementary Figure 2B**). However, a significant upregulation in MMP-9 and -11 mRNA expression was noted in Type 2 tumors compared to normal/benign tissues (**Figure 5B**). Overall, MMP-9, -11 and -14, but not -2, mRNA increased with Stage or WHO classification, with mRNA of MMP-11 being expressed several-fold higher than of the other MMPs.

### 3.4 Levels of TIMPs in the Ascites of High-Grade Ovarian Cancer Patients and in Patients Before and After Chemotherapy Treatments

Ascites collected from patients prior to debulking surgery and chemotherapy treatment, is termed as chemo-naïve (CN), and from patients collected at the time of disease relapse after undergoing chemotherapy treatment, is termed chemoresistant (CR).

**Figure 6:**
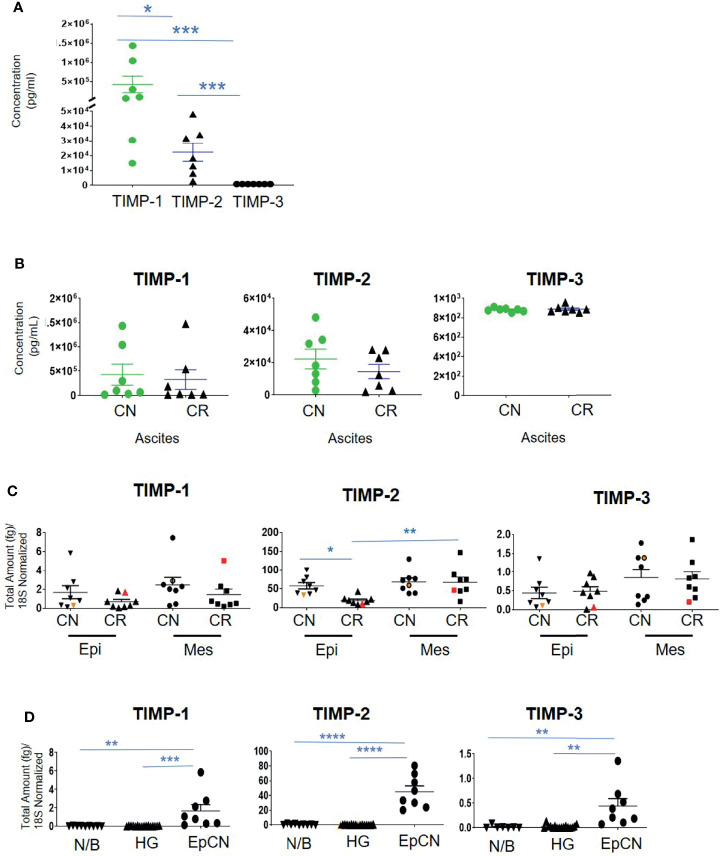
The concentration of TIMPs in the ascites of ovarian cancer patients. Graphs represents **(A)** the observed concentrations (pg/mL) of TIMP proteins in ascites from CN (n=7) patients; **(B)**, concentration of TIMP-1, -2 and -3 in CR (n=7) and CN (n=7) ascites of patients with serous ovarian cancer. Values are mean ± SEM; significance is determined by One-way ANOVA (Tukey’s multiple comparison test) and indicated by *p<0.05, ***p<0.001; **(C)** Expression of TIMPs mRNAs in ascites-derived epithelial (Ep) and mesenchymal (Mes) cells from CN and CR patients; **(D)** mRNA Expression of TIMPs in different ovarian tissues. Graphs represents total mRNA amounts (fg) of TIMP-1, -2 and -3 normalized to total amount of 18S, from benign/normal ovarian tissue (N/B), high-grade primary tumors (HG) and epithelial cells collected from CN ascites (EpCN). Values are mean ± SEM; significance was determined by One-way ANOVA (Tukey’s multiple comparison test) and indicated by *p<0.05, **p<0.01, ***p<0.001 and ****p<0.0001.

Contrary to the immunohistochemistry expression of TIMP-1 in ovarian tumors, the concentration of TIMP-1, measured by ELISA, was much higher than the concentrations of TIMP-2 and TIMP-3 (***p>0.0001 vs TIMP-3; *p>0.05 vs TIMP-2) in the ascites of CN patients (**Figure 6A**). In the ascites of CN patients, the concentration of TIMP-1 was 19-fold more than TIMP-2, and >482-fold higher than TIMP-3 (**Figure 6A**). In addition, the concentration of TIMP-2 was significantly higher than the concentration of TIMP-3 in CN ascites (***p>0.001) (**Figure 6A**). However, no significant difference was observed in the TIMP levels in ascites between CN and CR patients (**Figure 6B**). Overall, the circulating protein levels of TIMP-1 and -2 in ascites were well in excess of TIMP-3, and were not influenced by chemotherapy.

### 3.5 Expression of mRNAs of TIMPs and MMPs in CN and CR Ascites-Derived Tumor Cells Before and After Chemotherapy Treatment of Patients

Ascites-derived epithelial and mesenchymal cells were collected as described previously (37). We have shown previously that the non-adherent epithelial population expressing CA-125 are tumorigenic, whereas the mesenchymal cells that mainly expressed fibroblast-activating protein (FAP) and were negative for CA-125 expression are non-tumorigenic (37). RNA was extracted from both the epithelial and mesenchymal populations of CN and CR ascites-derived cells and absolute mRNA expression of TIMP-1, -2 and -3 were quantified by qRT-PCR. The epithelial and mesenchymal populations did not show any significant differences in the mRNA expression of TIMP-1 and TIMP-3 between the CN and CR patients (**Figure 6C**). On the other hand, TIMP-2 mRNA expression was significantly downregulated in the epithelial ascites-derived CR cells compared to the same population of cells in CN patients (**Figure 6C**). In addition, when comparing changes within the CR patients, TIMP-2 mRNA expression was significantly higher in the mesenchymal population of relapsing CR patients compared to the epithelial population (**Figure 6C**). In addition, there was a significantly higher intracellular mRNA expression of TIMP-1, -2 and -3 in CN ascites-epithelial or tumorigenic cells compared to either benign or primary ovarian tumors (**Figure 6D**). Mesenchymal or non-tumorigenic derived ascites cell population was not used for comparison. Interestingly, in both **Figures 6C, D** the TIMP-2 mRNA expression was much higher than both TIMP-1 and TIMP-3.

**Figure 7:**
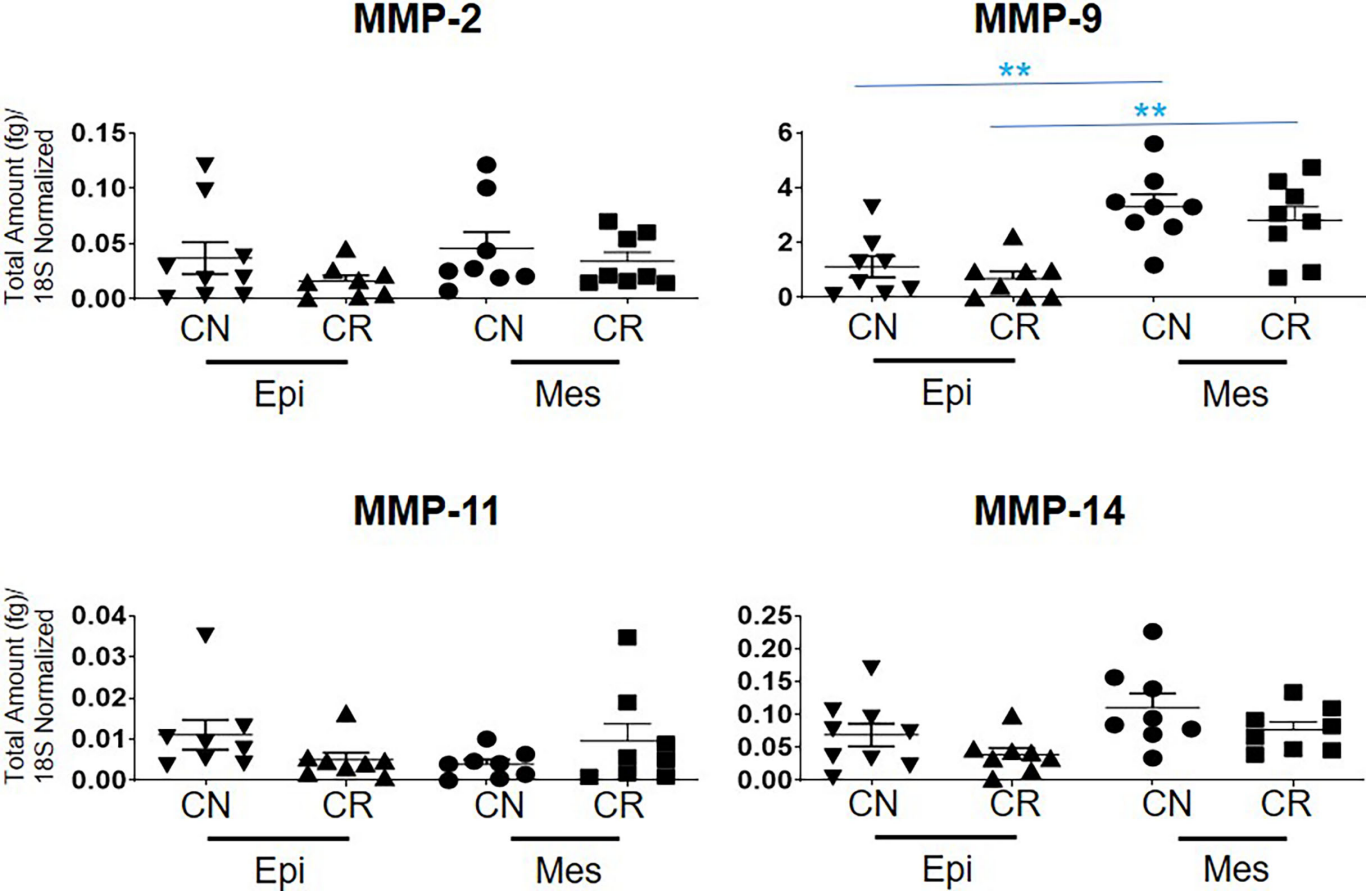
mRNA expression of MMP genes in ascites-derived epithelial (Ep) and mesenchymal (Mes) cells from CN and CR patients. Graphs represents the MMP-2, -9, -11 and -14 mRNA expression across these samples. The total mRNA expression (fg) was normalized to 18S in these samples. Values are mean ± SEM; significance was determined by One-way ANOVA (Tukey’s multiple comparison test) and is indicated by **p < 0.001.

The mRNA expression of MMP-2, -11 and -14 showed no significant differences between the epithelial and mesenchymal populations in CN and CR patient groups (**Figure 7**). However, a significant upregulation (**p<0.01 and *** p<0.001) of MMP-9 mRNA was noted in the mesenchymal compared to epithelial cells in both CN and CR groups (**Figure 7**).

### 3.6 mRNA and Protein Expression of TIMPs and MMPs in Ovarian Cancer Cell Lines

**Figure 8:**
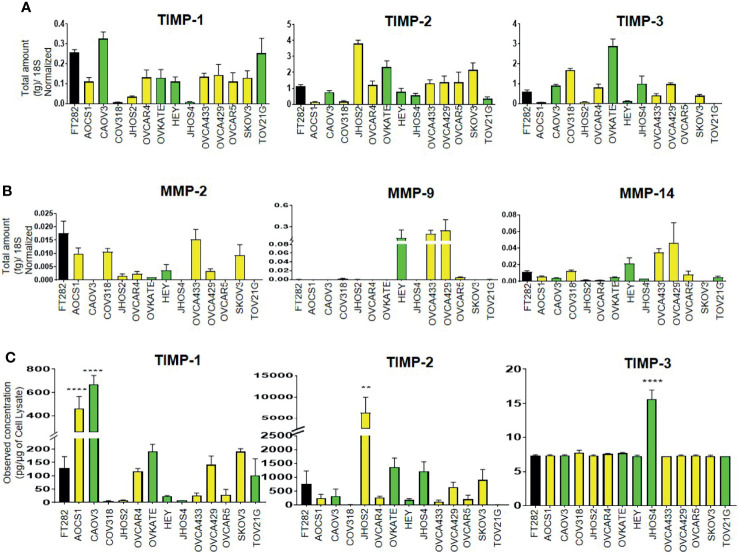
Differential mRNA expression of **(A)** TIMPs and **(B)** MMPs in ovarian cancer and FT282 cell lines deduced by qRT-PCR as described in *Methods*. The bar graphs are mean ± SEM (fg) (n=3) for each cell line collected at different split passages. **(C)** The bar graphs represent total TIMP-1, TIMP-2 and TIMP-3 protein expression (pg/µg of cell lysate) in the cell lysates of FT282 and ovarian cancer cell lines. Values are mean ± SEM (n=3) for each cell line collected at different split passages. Significance was obtained using One-way ANOVA (Dunnett’s multiple comparison test), **p<0.01, ****p<0.0001 when compared to the control cell line FT282. Black bar indicates the normal epithelial cell line FT282, yellow bars indicate cell lines derived from ascites and green bars cell lines derived from primary tumors.

We screened a diverse range of ovarian cancer cell lines originating either from high-grade tumors (CAOV3, HEY, JHOS2, JHOS4, OVKATE and TOV21G); or ascites (SKOV3, OVCAR5, OVCAR4, AOCS1, CAOV318, OVCA433 and OVCA429) of ovarian cancer patients; and a normal Fallopian tube cell line (FT282) (48, 49) for the mRNA expression of TIMP-1, -2 and -3 and MMP-2, -9 and -14. TIMP mRNAs were differentially expressed across the different ovarian cancer cell lines and FT282 (**Figure 8A**). Interestingly, there was several-fold more mRNA expression of TIMP-2 and -3 than TIMP-1 in the cell lines. MMP-2 and -14 were also differentially expressed between the ovarian cancer cell lines; but MMP-9 was expressed in HEY, OVCA433 and OVC429, and was detectable in OVCAR5 and CAOV318 cell lines and was not detected in the other cell lines (**Figure 8B**).

Overall, the cell lines originating from primary tumors or ascites did not follow any particular pattern of high or low mRNA expression of TIMPs and MMPs, but showed similar patterns of variability in terms of both high and low expression of TIMPs and MMPs. Hence, it can be deduced that there is no correlation between the mRNA expression of TIMPs and MMPs with the origin (tumor or ascites) of ovarian cancer cell lines. When detectable in a cell line, TIMP-1 mRNA expression was much less than the other two TIMP mRNAs, and MMP-9 mRNA expression in three cell lines was greater than the other MMP mRNAs.

The protein levels of TIMPs, in the cell lysates of the Fallopian tube-derived control FT282 cell line and the cancer cell lines was measured by the ELISA assay as described in the *Methods* section. The protein levels of TIMPs were differentially expressed across all the cell lines studied (**Figure 8C**). TIMP-2 protein was highly expressed in JHOS2 with low expression in OVCA433, OVCAR4, OVCAR5, AOCS1 and COAV3 cell lines. Expression of TIMP-2 was below the level of detection in CAOV318 and TOV21G cell lines. Contrary to the mRNA results, TIMP-3 protein levels were extremely low to almost non-detectable across the cell lines with the exception of JHOS4 where it was slightly more expressed. TIMP-1 protein reflected TIMP-1 mRNA expression in CAOV318, JHOS2 and JHOS4 cell lines having the lowest expression, and CAOV3 the highest. TIMP-2 protein reflected TIMP-2 mRNA with CAOV318 and TOV21G cell lines having the lowest expression and JHOS2 the highest.

### 3.7 Expression of TIMPs, MMPs and Cancer Stem Cell (CSC) Markers in Different Ovarian Cancer Cell Lines in Response to Chemotherapy Treatment

**Figure 9:**
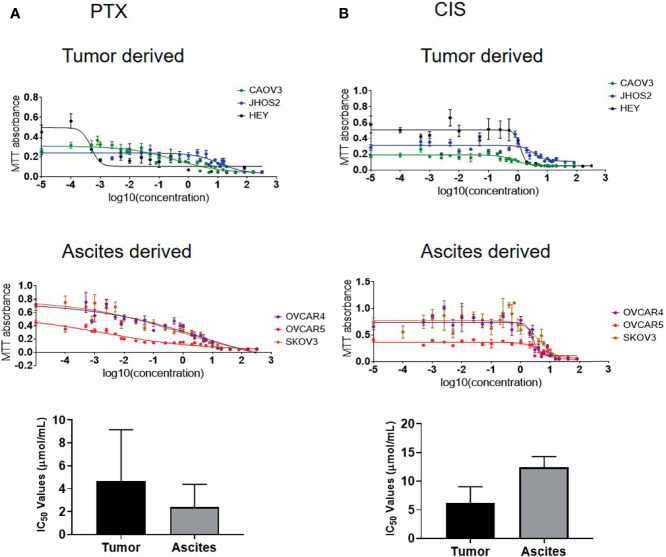
IC_50_ values of ovarian cancer cell lines in response to paclitaxel (PTX) and cisplatin (CIS) treatment. **(A)** IC_50_ values of tumor-derived CAOV3, JHOS2 and HEY and ascites-derived OVCAR4, OVCAR5 and SKOV3 cell lines treated with PTX with corresponding graph showing differences in the tumor and ascites-derived cell lines. **(B)** IC_50_ values of tumor-derived CAOV3, JHOS2 and HEY and ascites-derived OVCAR4, OVCAR5 and SKOV3 treated with CIS with corresponding graph showing differences in the tumor and ascites-derived cell lines. Cell lines were treated for 48 hours with varying concentrations (0 to 320 µg/ml) of either CIS or PTX and their IC_50_ values (the concentration that kills 50% of the cells) was determined by MTT assay. Data are representative of three independent experiments using cells at different passages treated in triplicate. Significance in bar graphs was determined by student’s t test (Mann-Whitney test).

Recent literature suggests that ovarian cancer cell lines derived from primary tumors are more likely to attach and invade than cell lines derived from ascites (50), indicating that tumor-derived cell lines may be more aggressive than those derived from ascites. The cell lines used in this study were derived from both ovarian tumors and ascites. To test if sensitivity to chemotherapy is related to the tissue of origin, three tumor-derived cell lines (CAOV3, JHOS2, and HEY) and three ascites-derived cell lines (OVCAR4, OVCAR5 and SKOV3) were tested for chemosensitivity against chemotherapy drugs cisplatin (CIS) and paclitaxel (PTX). In response to CIS treatment, the IC_50_ (50% growth inhibition) value of the tumor-derived cell lines was [CAOV3 (2.67 μmol/ml), JHOS2 (11.86μmol/ml), and HEY (3.97 μmol/ml)](**Figure 9** and **Supplementary Table 4**). However, the sensitivity to PTX in tumor-derived cells varied within a large range, with JOSH2 (13.62 μmol/ml) having the highest and HEY cells (0.47 mol/ml) having the lowest and COAV3 in between (0.41 μmol/ml) (**Figure 9** and **Supplementary Table 4**). On the other hand, in ascites-derived cells sensitivity to CIS was [OVCAR4 (8.73 μmol/ml, OVCAR5 15.23μmol/ml and SKOV3 (13.16 μmol/ml]. Similar to tumor-derived cells, sensitivity to PTX was 2.39 nmol/ml in OVCAR5, 6.36 µmol/ml in OVCAR4 cells and 0.80 μmol/ml in SKOV3. There was no significant difference in sensentivity towards CIS or PTX between tumor- or ascites-derived cell lines (**Figure 9** and **Supplementary Table 4**). It should however, be noted that OVCAR4 and OVCAR5 are derived from the ascites of different patients, with OVCAR5 derived from a chemo-naïve, while OVCAR4 from a patient after chemotherapy treatment (51–53).

**Figure 10:**
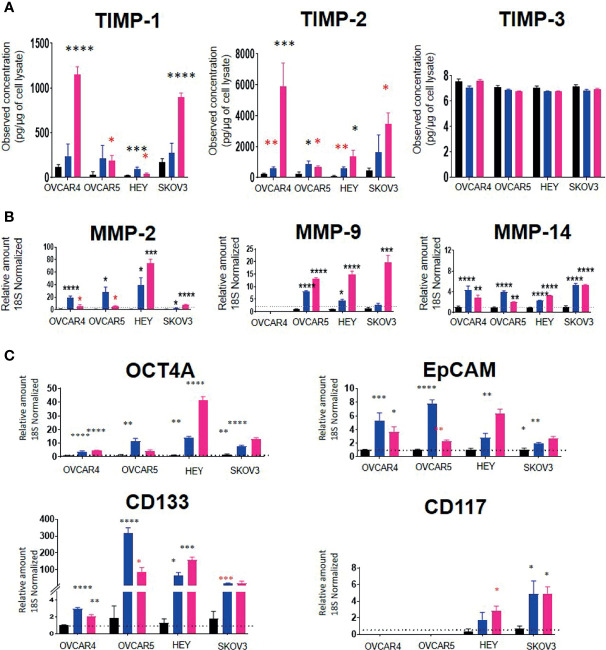
**(A)** TIMP protein levels in response to PTX and CIS treatments in ovarian cancer cell lines. OVCAR4, OVCAR5, HEY and SKOV3 cells lines were treated for 48 hours with their respective IC_50_ concentrations of CIS (blue bars) and PTX (pink bars). Black bars indicate the control untreated cell lines. The total TIMP-1, -2 and -3 protein expression (pg/µg of cell lysate) was deduced by ELISA as described in *Methods*. Values are mean ± SEM (n=3) for each cell line collected at different cell passages. **(B)** The mRNA expression of MMPs in CIS and PTX treated ovarian cancer cell lines. OVCAR4, OVCAR5, HEY and SKOV3 cells lines were treated for 48 hours with their respective IC_50_ concentrations of CIS (blue bars) and PTX (pink bars). The mRNA levels of MMP-2, -9 and -14 were obtained by relative qRT-PCR. Each experiment was repeated three times in triplicate. **(C)** mRNA expression of CSC marker genes in ovarian cancer cell lines treated with CIS and PTX. OVCAR4, OVCAR5, HEY and SKOV3 cells lines were treated for 48 hours with their respective IC_50_ concentrations of CIS (blue bars) and PTX (pink bars). Black bar indicates the control untreated cell lines. The mRNA expression levels of chemoresistance associated stem cell markers were measured by relative qRT-PCR. Each experiment was repeated three times in triplicate. Significance (black asterix) was determined by one-way ANOVA (Dunnett’s multiple comparison test) *p>0.05; **p>0.01; ***p>0.001; ****p<0.0001 compared to control (untreated parental). Significance (red asterix) *p>0.05; **p>0.01 was determined by Student t-test compared to control.

Using these IC_50_ doses of chemotherapeutics, we then investigated the corresponding TIMP-1, -2 and -3 protein levels in cell lysates from selected control vs CIS and PTX- treated cell lines. TIMP-1 protein in the cell lysates was increased significantly by PTX in all four cell lines tested (OVCAR4, OVCAR5, HEY and SKOV3) (**Figure 10A**). However, TIMP-1 was only significantly upregulated by CIS treatment in the HEY cell line (**Figure 10A**). There was a significant upregulation of TIMP-2 after CIS and PTX treatment in the OVCAR4, OVCAR5 and HEY cell lines (**Figure 10A**). However, TIMP-2 protein was only significantly upregulated in the SKOV3 cell line after PTX. TIMP-3, on the other hand, was expressed at relatively low levels in all the cell lysates compared to other TIMPs (**Figure 10A**).

MMP-2 and -14 mRNA levels were significantly upregulated by both CIS and PTX treatments in all four-cell lines (**Figure 10B**). However, MMP-9 mRNA was upregulated by both treatments in OVCAR5 and HEY cell lines only. PTX alone significantly, upregulated MMP-9 mRNA in SKOV3 cells (**Figure 10B**). There was no MMP-9 mRNA expression detected in OVCAR4 cells before or after chemotherapy treatment. mRNA expression of TIMPs and MMPs with IC_50_ values of PTX and CIS in ovarian cancer cell lines has been described in **Supplementary Table 4**.

To test if the cell lines treated with chemotherapy drugs exhibited a chemoresistant phenotype associated with an enhanced expression of CSCs markers compared to control untreated cells, the mRNA expression of the CSC markers, OCT4A, EpCAM, CD133 and CD117, was performed (**Figure 10C**). CIS and PTX treatment enhanced the mRNA of CSCs markers (**Figure 10C**). OCT4A, EpCAM and CD133 mRNA levels were upregulated in all four-cell lines by both chemotherapies. CD117 mRNA was not expressed in OVCAR4 or OVCAR5 cells and was upregulated by both chemotherapies in HEY and SKOV3 cells only.

### 3.8 TIMPs, MMPs and the TCGA Data Set

**Figure 11:**
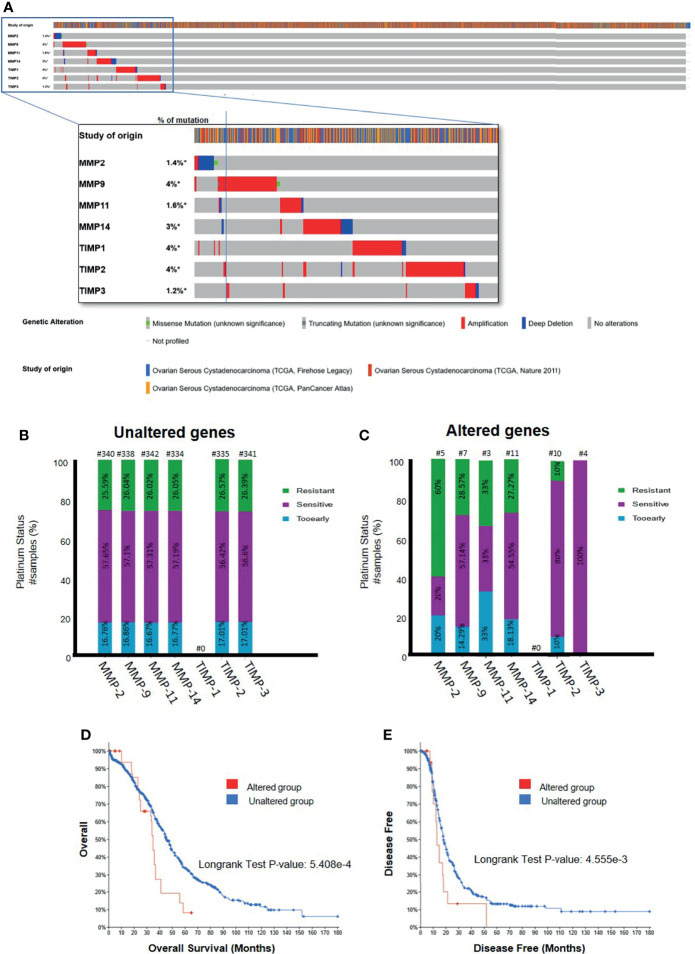
**(A)** Mutational Oncoprint results for MMP and TIMP genes from data in the cBioPortal for Cancer Genomics (TCGA data set). Data is from 3 original studies totaling 1680 samples (606 in the Firehose Legacy study, 489 in the Nature 211 study, and 585 in the PanCancer Atlas study). The colored bands in the horizontal line at the top represent the study of origin. Each vertical line in that band indicates a single sample and the horizontal bands below correspond to the TIMP and MMP genes analyzed in those vertical samples. Percentages indicate the mutational percentage of that gene in the total samples studied. Red, blue, green and grey indicate the type of genetic alteration in each sample. **(B,C)** Platinum Status of patients in relation to MMP and TIMP genes; **(B)** patients with unaltered genetic mutation; and **(C)** patients with genes altered by mutation. The data is based on 1680 samples as described in **(A)**. Percentage of patients are indicated in each of the histograms. Above each bar, the number of patients is indicated (#). **(D, E)** Overall and disease-free survival curves of patients with MMP-14 gene expression. Data as described above querying 1668 patients/1680 samples for the MMP-14 gene. **(D)** Overall survival data comparing the altered versus wild type MMP-14 gene. **(E)** Disease free survival for patients with altered versus unaltered mutation of the MMP-14 gene. Significance indicated using the Logrank Test as indicated in the graphs.

Interrogation of three datasets on ovarian serous cystadenocarcinomas (TCGA Firehose Legacy, TCGA Pan Cancer Atlas, TCGA Nature 2011) [http://www.cbioportal.org] comprising 1680 samples showed a very small percentage of genetic alteration (1.2-4%) of TIMP-1, -2, -3 and MMP-2, -9, -11 and -14 genes (**Figure 11A**). Most of the genetic alterations occurred through gene amplification followed by deep deletion, whereas there were minuscule numbers with missense mutations (**Figure 11A**). Further interrogation of the data to determine the platinum response in patients in the above three cohorts showed that 57% of the patients who had unaltered TIMP-2, -3 and MMP-2, -9, -11 and -14 genes were sensitive to platinum, while ~27% were resistant to platinum therapy (**Figure 11B**). On the other hand, genetic alteration of MMP-2 and -11 genes shifted the number of patients resistant to platinum therapy to an increased level of 60 and 33%, respectively (**Figure 11C**). Contrary to that, alteration in TIMP-2 and -3 genes increased the number of patients sensitive to platinum to 80 and 100%, respectively (**Figure 11C**). These studies suggest that alterations in genes (mostly amplification by copy number of genes, RNA or protein) coding for TIMPs and MMPs, even though in low numbers, changes the status of the platinum response in ovarian cancer patients; more becoming sensitive to platinum therapy with TIMP-2 and -3 mutations while this is reversed for MMP-2 and -11 mutations. Further examination of the data suggested that alteration in gene expression of MMP-14 in ovarian tumors decreased the OS and PFS in patients compared to those who had unaltered MMP-14 gene (**Figures 11D, E**). However, no such affect was observed for TIMP-2, -3 and MMP-2, -9, -11 genes (**Supplementary Table 5**). The TCGA dataset lacks information on the platinum response in relation to TIMP-1 gene.

## 4 Discussion

TIMPs and their associated MMPs are active regulators of ECM remodeling and hence, are crucial for tumor progression (54–56). Even though their expression patterns in ovarian tumors have been described previously, it is still ambiguous requiring further investigation (57–59). In addition, very little data is available on how these proteins are regulated by chemotherapy treatment in patients. The aim of this study was to extensively determine the expression of TIMPs and the related MMPs in epithelial serous ovarian tumors, ascites, ascites-derived tumor cells obtained from patients before (CN) and after chemotherapy treatments and at recurrence (CR), and in ovarian cancer cell lines before and after chemotherapy treatment. A further aim was to align our findings with the publicly available TCGA dataset to establish the expression of TIMPs and MMPs in ovarian cancer progression and after chemotherapy treatment. Overall, our results show that: (i) the immunohistochemistry expression of TIMP-1 was low compared to the levels of TIMP-2 and -3 in serous ovarian tumors and no significant differences could be observed between different Stages, Grades and Type of tumors compared to control. (ii) The levels of TIMP-2 and -3 increased with WHO classification of ovarian tumors particularly, with Type 2 ovarian tumors, showing greater intensity of staining of TIMP-2 than -3 relative to control. (iii) MMP-9, -11 and -14, but not MMP-2, mRNA expression increased with Stage and WHO classification compared to control, with MMP-11 being expressed in more abundance than other MMPs. (iv) The protein levels of TIMP-1 and -2 were well in excess of TIMP-3 in the ascites of CN patients, and were not influenced by chemotherapy. (v) Further to that, intracellular mRNA levels of TIMP-2 was significantly higher in the tumorigenic epithelial cells of CN patients compared to CR patients; and TIMP-2 was significantly high in the fibroblast-like mesenchymal CR cells compared to epithelial CR cells. (vi) Despite a large variation in the levels of TIMPs and MMPs mRNA levels in ovarian cancer cell lines, no correlation could be deduced with the origin (primary tumor or ascites) of the cell lines. When detectable in a cell line, TIMP-1 mRNA expression was much less, than the other two TIMP mRNAs, and MMP-9 mRNA expression in the cell lines studied was greater than MMP-2, -11 and -14 mRNAs. There was a large variation in the level of detectable TIMP protein expression in the fourteen cell lines studied, with TIMP-1 and -2 being well in excess of TIMP-3 levels. (vii) PTX treatment significantly, and to a much lesser extent (non-significantly), CIS treatment, increased the levels of TIMPs -1 and - 2, but not TIMP-3, in cell lysates. With the exception of MMP-9 mRNA in OVCAR4 cells, CIS and PTX both significantly increased the expression of MMP-2, -9 and -14 mRNAs and concurrently increased the expression of three CSC markers (OCT4A, EpCAM, CD133), associated with chemoresistance in the cancer cell lines. (viii) Finally, the TCGA data sets suggests, the platinum response in ovarian cancer patients was related to the amplification of the TIMP and MMP genes; patients becoming sensitive to chemotherapy with TIMP mutations and resistant with MMP mutations.

The strength of this study is immunohistochemistry-based localization of TIMP-1, -2 and -3 coinciding with the expression of CA-125 on the same ovarian tumor sections, showing positive localization of TIMPs in the actual tumor cells. Previous studies embracing the co-localization of TIMPs with CA-125 have not been reported. Even though, in our study the immunohistochemistry detection of TIMP-2 was not significantly increased by FIGO Stages, the expression of TIMP-3 did go up significantly compared to normal/benign tissues. A lack of significance in TIMP-2 expression in relation to increasing Stages may be due to the low enhanced increment of TIMP-2 expression in a relatively low number of samples in each FIGO Stage [seen particularly in FIGO II (only 4 samples) and FIGO IV (only 2 samples)]. As a result, a statistically significant increase in TIMP-2 expression with respect to increasing Stages compared to normal/benign tissues was not attained. However, with TIMP-3 staining a relatively higher expression level in each FIGO Stage provided a significant difference in the expression level with respect to Stages. Nonetheless, a significant upregulation in the expression of TIMP-2 expression according to WHO classification was consistent with TIMP-3 expression. Previous studies have reported expression of TIMP-2 in the stroma of tumors and positively correlated that with better survival rate in ovarian cancer patients (59). Contrary to that, an increase in epithelial and stromal TIMP-2 expression was shown to have no correlation with patient death/survival or with tumor progression in patients with advanced ovarian cancer (57, 58). Even though these studies used immunohistochemistry of tumor sections, none used a marker to identify tumor areas, but relied on automated analytical systems that provided the overall expression of TIMP-2 in tumors, which may not correspond to positive neoplastic cells.

Contrary to our results above, other groups have reported high expression levels of TIMP-1 in Stages III and IV correlating with decreased overall survival in patients (60). This particular study noted enhanced mRNA expression of TIMP-1 in high-grade ovarian tumors obtained from KM-plotter database. However, we show low expression of TIMP-1 in ovarian tumors compared to the expression of TIMP-2 and -3. Nevertheless, several fold higher expression of TIMP-1 was noted in the ascites of CN patients compared to TIMP-2 and -3. This may suggest that TIMP-1 in ovarian cancer may well be in the tumor secretome in contrast to being retained in the tissues. In that context, molecular characterization of epithelial circulating cells in patients with disseminated ovarian cancer has shown significantly high expression of TIMP-1 suggesting the utility of TIMP-1 as a biomarker for disseminated disease (61).

Significantly, higher expression of TIMP-2 in Type-2 compared to Type-1 tumors supports the involvement of this protein in tumor progression as well as treatment outcome. In that context, a recent study using bioinformatics has reported TIMP-2 as a poor prognostic indicator in gastric cancer (62); in addition, TIMP-2 has been shown to regulate matrisome-associated genes in cancer (54). In the same context, upregulation of TIMP-3 expression in Stage III, Grade 3 and Type 2 tumors deduced from this study, points to the involvement of TIMP-3 in tumor progression and treatment outcome. This however, is in contrast to the well-documented tumor-suppressor role of TIMP-3 (63).

We also report a significant increase in the mRNA expression of MMP-9, -11 and -14 in Stage IV and in Type 2 tumors compared to normal/benign samples may indicate changes in ECM modelling as a prerequisite for cancer progression. An increase in MMP-11 in cancers has been associated with increased proliferation and poor disease outcome in patients (64, 65). While high MMP-2 and MMP-9 expression have been associated previously with neo-angiogenesis and tumor vascularization (66), an increase in protein expression of MMP-9 correlated with an increase in patient’s death rate diagnosed with high-grade serous ovarian cancer (58). In addition, the membrane bound MMP-14 has been linked to specific roles in ovarian cancer cell-matrix detachment, migration, ECM invasion and angiogenesis (67, 68). MMP-14 is crucial for the shedding of ovarian cancer cells from primary tumors into the peritoneal cavity where they accumulate as multicellular aggregates for stromal invasion and further dissemination (69). In addition, active MMP-14 promotes ectodomain shedding of MUC16/CA125 in ovarian cancer cells, restrains adhesion and promotes invasion of cancer cells in the peritoneum (70). Thus, the upregulated MMPs observed in advanced-stage ovarian carcinomas may be vital in aiding progression of cancer (71). However, the concurrent enhanced expression of TIMP-2, -3 and MMP-9, -11, -14 observed in ovarian carcinomas is conflicting with the potential inhibitory actions of the TIMPs on MMPs and requires further investigation (72).

As ascites is a major facilitator of ovarian cancer peritoneal dissemination in 40% of patients, we investigated the expression of TIMPs and MMPs in ascites and ascites-derived tumor epithelial and stromal mesenchymal cell populations from CN and CR patients. We report for the first time the greater abundance of TIMP-1 in CN ascites compared to TIMP-2, which again was in excess to TIMP-3 levels. Low expression of TIMP-3 in ascites is consistent with its characteristics of being ECM bound while TIMP-1 and -2 being more secretory in the ECM space (73, 74).

At the cellular level in ascites, a significant decrease in TIMP-2 mRNA expression in the ascites-derived epithelial tumorigenic CR cells compared to CN cells may occur to foster a more aggressive phenotype in floating ascites-derived tumorigenic cells through MMPs for the dissemination of malignant cells during cancer progression (21). In addition, the low level of TIMP-2 mRNA in the floating CR cells in the ascites of relapsed patients may also indicate a nonexistent requirement for matrisome-associated gene expression due to their prolonged floating status (54). This is consistent with low expression of certain plakins and integrins in CR versus CN ascites-derived tumor cells reported by us previously (75, 76). We further show that even though there is no difference in the mRNA expression of TIMP-2 between Mes (CN) and Mes (CR) ascites-derived cells, a significant enhanced mRNA expression of TIMP-2 was observed within the CR cohort of Mes-stromal compared to Epi-tumorigenic population. This again indicates that in CR patients TIMP-2 expression is more relevant to stromal cells, which are attached to the matrix (adherent population) (37), again indicating a crucial role of TIMP-2 in the regulation of ECM matrisomes.

We also report a significant upregulated mRNA expression of MMP-9 in the mesenchymal compared to epithelial cells in CR patients. In that context, an elevated MMP-9 mRNA in mesenchymal cells of CR patients, correlating with a decrease in E-cadherin expression was reported by us previously (37), consistent with the role of MMP-9 in epithelial-mesenchymal transition (EMT) in platinum resistant ovarian and other cancers (77, 78). We have also reported previously an enhanced secretion of MMP-2 and MMP-9 by floating ovarian cancer spheroids, with diminished expression of several integrins (79).

In this study, we also show that cisplatin (CIS) and paclitaxel (PTX) enhanced the expression of TIMP-1 and TIMP-2 at the protein level in all four ovarian cancer cell lines studied. However, TIMP-3 protein remained unchanged in all four-cell lines studied. The upregulation of TIMP-1 and -2 in response to chemotherapy treatment was consistent with the upregulation of mRNA levels of MMP-2, -11 and -14. In addition, after chemotherapy treatment the cell lines also exhibited a chemoresistant phenotype as exhibited by the enhanced expression of CSC markers as described previously (39, 80, 81). The above data may suggest chemo protective roles of TIMP-1, -2, and MMP-2, -11 and -14 in chemotherapy surviving ovarian cancer cells. Chemotherapy-induced changes in ECM proteins that provide chemo protective effects on chemotherapy surviving cells have been shown (82, 83). Up regulation of TIMPs and other MMPs in response to cytotoxic drugs on cells grown under adherent conditions may indicate reorganization of the ECM in response to the cytotoxic effects of chemotherapy, which reprograms the surviving cells into a distinct phenotype (77).

Current literature suggests a chemo protective role of TIMPs mediated through tumor-induced MMP-independent regulation (84). An upregulation of TIMP-1 in breast cancer was associated with poor patient response to chemotherapy, due to TIMP-1 providing protection against apoptosis (85). In addition, TIMP-1 overexpressed and secreted by platinum resistant ovarian cancer altered migration of cancer cells and facilitated the growth of endothelial cells (60). In that connection, we previously reported and report in this study upregulation of TIMP-2 mRNA and protein levels in response to chemotherapy treatment in ovarian cancer cell lines (23). We have also reported that ovarian cancer cells can be sensitized to chemotherapy by suppressing the expression of TIMP-2 (38). Our previous studies have shown activation of Stat3 is critical for the development of chemoresistance in ovarian cancer cells (39, 81). In that context, chemotherapy treatment of ovarian cancer cells in which TIMP-2 was knocked down failed to activate Stat3, implying that TIMP-2 and Stat3 may be critical regulators of chemoresistance (38).

The status of MMPs in response to chemotherapy treatment is conflicting in current literature. For example, MMP-2 mRNA expression was significantly higher in ovarian cancer cells that was sensitive to chemotherapy compared with resistant/refractory tumors, while no significant change in MMP-9 mRNA expression was noted (86). While in osteosarcoma, a predominant MMP-2/MMP-9 activity is associated with poor response to chemotherapy (87). Since a major role of TIMPs is to inhibit MMP activities, it is expected that upregulation of TIMP expression after chemotherapy treatments may result in decreased MMP-2/MMP-9 expression. Upregulation of both TIMPs and MMPs in response to chemotherapy treatment may indicate TIMP-independent regulatory roles of MMPs described in the literature (88, 89).

In this study, we aligned observations on TIMPs and MMPs obtained from patients ‘samples and in *in vitro* chemotherapy treated cancer cell lines with the data available in public domain (TCGA). Even though the TCGA datasets from three studies on 1680 serous ovarian carcinomas suggests a prognostic utility (OS and PFS) of MMP-14 based on its altered and unaltered genetic expression in serous ovarian cancer patients, no such links could be made with TIMP-1, -3 or MMP-2, -9, and -11. However, limited alteration in TIMP gene amplifications, deep deletion or missense mutations changed platinum responses in ovarian cancer patients, with the majority becoming chemosensitive with alterations in TIMP-2, or -3 gene mutations. Contrary to that, small number of mutations in MMP-2 and -11 genes increased the percentage of platinum resistant patients, suggesting that genetic alterations in TIMPs and MMPs shifts the platinum response status in ovarian cancer patients.

In summary, this is the first study that analyzed TIMPs and associated MMPs in a range of ovarian cancer patient samples and in cell lines with and without chemotherapy treatment. The findings that TIMPs and MMPs promote ovarian cancer progression and chemoresistance is clinically relevant as demonstrated by evaluation of patients’ samples, *in vitro* cell line studies and TCGA datasets. Even though a lot of work remains in understanding the roles of TIMPs and MMPs in ovarian cancer progression and chemoresistance, this study highlights potential new targets for intervention to overcome chemoresistance in ovarian cancer patients.

## Data Availability Statement

The original contributions presented in the study are included in the article/**Supplementary Material**. Further inquiries can be directed to the corresponding author.

## Ethics Statement

This project (Project 09/09) was approved by the Research and Ethics Committee of Royal Women’s Hospital (RWH), Melbourne, Australia. The patients/participants provided their written informed consent to participate in this study.

## Author Contributions

RE contributed to data collection and analysis, conceptualization of the data, writing and editing of the manuscript. GK was involved with the editing of the manuscript. JF was involved with the conceptualization of data, design of the study and edited the manuscript. NA conceived the idea, designed, wrote and edited the manuscript. All authors have read and approved the manuscript.

## Funding

This work was supported by the John Turner Cancer Research Funds to Fiona Elsey Cancer Research Institute, Ballarat, Australia. RE is a recipient of Australian postgraduate award.

## Conflict of Interest

The authors declare that the research was conducted in the absence of any commercial or financial relationships that could be construed as a potential conflict of interest.

## Publisher’s Note

All claims expressed in this article are solely those of the authors and do not necessarily represent those of their affiliated organizations, or those of the publisher, the editors and the reviewers. Any product that may be evaluated in this article, or claim that may be made by its manufacturer, is not guaranteed or endorsed by the publisher.

## Supplementary Material

Supplementary Figure 1 TIMP-1 expression in primary ovarian tumors according to different tumor classifications. Representative images of TIMP-1 staining in primary ovarian tumors (n=26) and benign ovarian samples (n=8). Samples were sorted into Stages, WHO classifications and Grades. Magnification 40X, scale-bar = 50uM. Red dots indicate patients carrying BRCA mutations and black/white dots indicate patients with ascites present at the time collection.

Supplementary Figure 2 mRNA expression in primary ovarian tumors. **(A)** TIMPs mRNA expression in primary ovarian tumors according to different tumor classification. Samples were stratified into FIGO Stages, Silverberg and WHO classifications. **(B)** MMPs mRNA expression in primary ovarian tumors according to Silverberg classification. Red dots indicate patients carrying BRCA mutations and black/white dots indicate patients with ascites at the time of collection. Values are mean ± SEM (n=3).
