# Supplementary figures and images for: Expression of TIMPs and MMPs in Ovarian Tumors, Ascites, Ascites-Derived Cells, and Cancer Cell Lines: Characteristic Modulatory Response Before and After Chemotherapy Treatment
Source: Front Oncol. 2022 Jan 3;11:796588. doi: 10.3389/fonc.2021.796588 (PMC8762252; doi:10.3389/fonc.2021.796588)

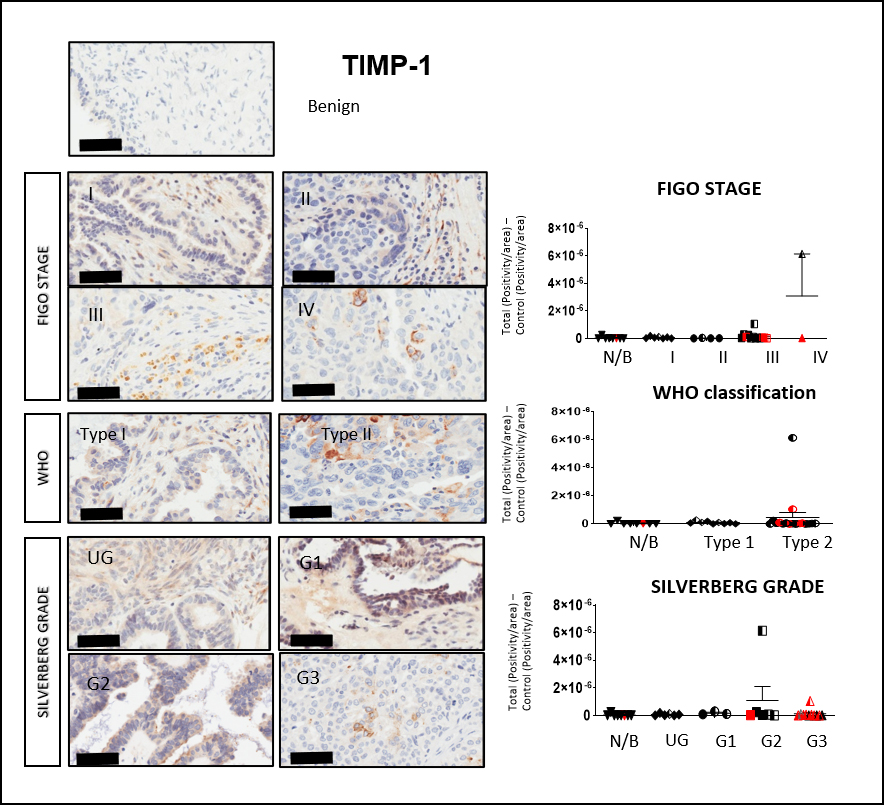

Supplement: Supplementary Figure 1 — TIMP-1 expression in primary ovarian tumors according to different tumor classifications. Representative images of TIMP-1 staining in primary ovarian tumors (n=26) and benign ovarian samples (n=8). Samples were sorted into Stages, WHO classifications and Grades. Magnification 40X, scale-bar = 50uM. Red dots indicate patients carrying BRCA mutations and black/white dots indicate patients with ascites present at the time collection. [file Image_1.jpeg]

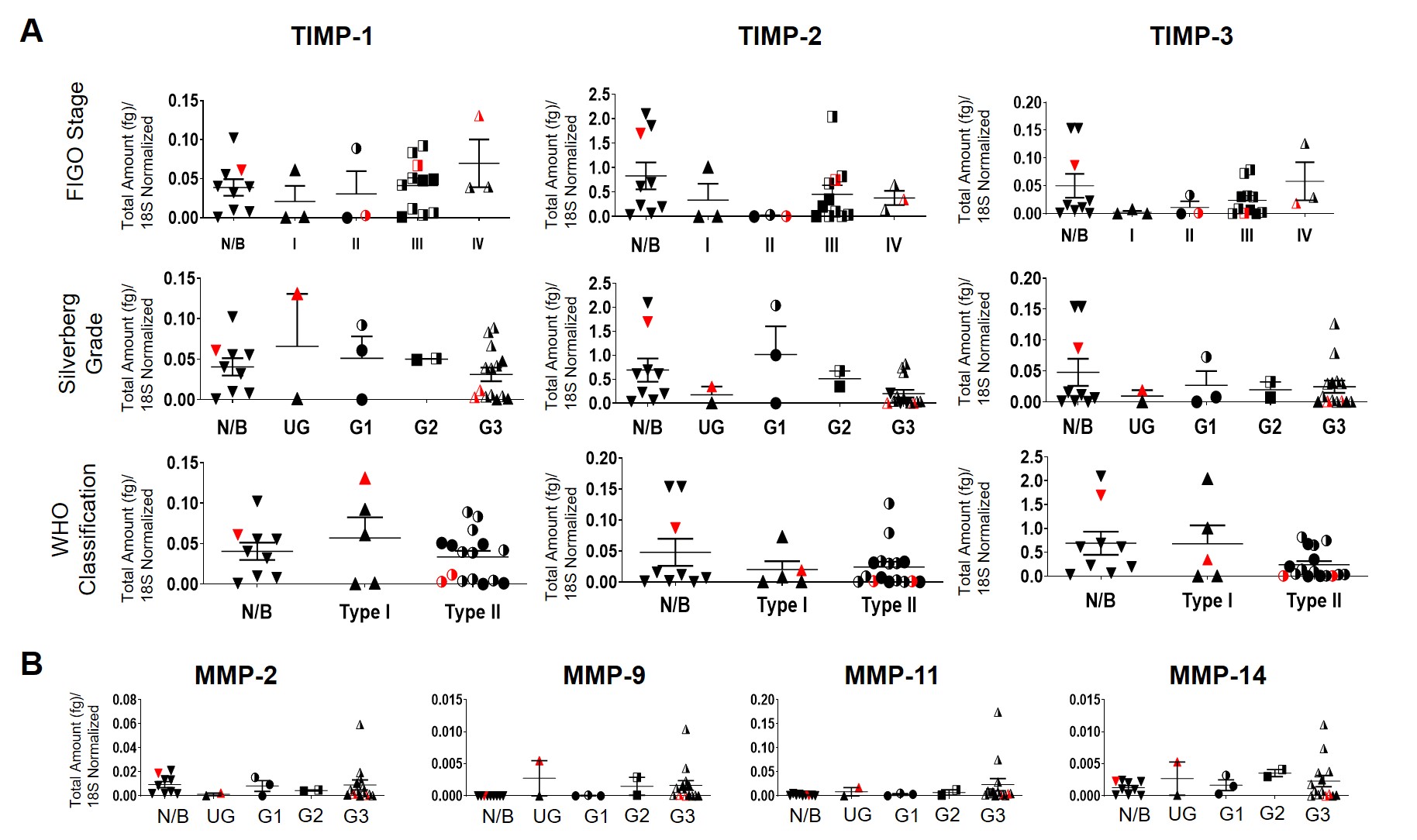

Supplement: Supplementary Figure 2 — mRNA expression in primary ovarian tumors. (A) TIMPs mRNA expression in primary ovarian tumors according to different tumor classification. Samples were stratified into FIGO Stages, Silverberg and WHO classifications. (B) MMPs mRNA expression in primary ovarian tumors according to Silverberg classification. Red dots indicate patients carrying BRCA mutations and black/white dots indicate patients with ascites at the time of collection. Values are mean ± SEM (n=3). [file Image_2.jpeg]
