# Supplementary material for: Expression of TIMPs and MMPs in Ovarian Tumors, Ascites, Ascites-Derived Cells, and Cancer Cell Lines: Characteristic Modulatory Response Before and After Chemotherapy Treatment
Source: Front Oncol. 2022 Jan 3;11:796588. doi: 10.3389/fonc.2021.796588 (PMC8762252; doi:10.3389/fonc.2021.796588)
Supplement: Supplementary file 3 [file Table_1.docx]

Supplementary Material

**Supplementary Table 1 (A): Clinical information of the patients recruited for immunohistochemistry study**

| Block # | Age | Pathologist diagnosis | Tumour Type | FIGO Stage | Silverberg Classification | WHO classification | Ascites Present at Diagnosis | Genetic Info. |
| --- | --- | --- | --- | --- | --- | --- | --- | --- |
| 2 | 20 | Benign sclerosis tumour | benign | -- | -- | -- | no | Nil Ca |
| 3 | 45 | normal OV and FT | normal | -- | -- | -- | no | BRCA1+ |
| 4 | 43 | benign serous cystadenoma (normal- Peutz-Jegher's Syndrome) | benign | -- | -- | -- | no | Peutz-Jegher's syndrome |
| 5 | 54 | Fibroma-mitotically active (benign) | benign | -- | -- | -- | no | Nil Ca |
| 6 | 64 | serous cystadenofibroma/ Large multi cystic ovarian mass (benign) | benign | -- | -- | -- | no | Family history of Ca |
| 7 | 58 | serous cystadeno-fibroma (Cyst benign) | benign | -- | -- | -- | no |  |
| 8 | 62 | serous cystadeno-fibroma/ Large ovarian cyst (benign) | benign | -- | -- | -- | no | n/a |
| 9 | 48 | simple serous cyst (benign) | benign | -- | -- | -- | no | HNPCC carrier (colorectal Ca) |
| 10 | 54 | serous cystadenoma – borderline malignancy | borderline | Ia | not graded | 1 (Low) | not entered | Nil Ca |
| 11 | 60 | serous cystadenoma borderline malignancy | borderline | Ia | not graded | 1 (Low) | no | Nil Ca |
| 12 | 37 | serous cystadenoma - borderline, microinvasion | malignant | Ia | not graded | 1 (Low) | no | no (grandfather Ca pancreas) |
| 13 | 49 | serous cystadenoma borderline malignancy | borderline | Ib | not graded | 1 (Low) | unknown | Nil Ca |
| 15 | 31 | micropapillary Ser Ca, Ser Cys NOS | malignant | Ic | G1 | 1 (Low) | no | grandmother gastric Ca grandfather smoker lung Ca |
| 16 | 67 | serous cystadenoma borderline malignancy | borderline | Ic | not graded | 1 (Low) | no | niece and nephew Bowel Ca |
| 17 | 44 | Papillary Ser Cys uncertain benign or malignant /serous borderline tumour | borderline | Ic | not graded | 1 (Low) | yes | n/a |
| 18 | 54 | Ser Ca, Papillary Ser Cys | malignant | IIc | G3 | 2 (High) | no | mother kidney Ca, Br Ca |
| 19 | 67 | Ser Ca, Papillary Ser Cys | malignant | IIc | G3 | 2 (High) | yes | sister died melanoma |
| 20 | 61 | Ser Ca, Ser Cys NOS | malignant | IIc | G3 | 2 (High) | no | Nil Ca |
| 21 | 72 | Ser Caꭞ, Papillary Ser Cys | malignant | IIc | G3 | 2 (High) | unknown | Nil Ca |
| 22 | 65 | Ser Ca, Ser Cys NOS | malignant | IIb | G2 | 2 (High) | no | Nil ca |
| 23 | 74 | Ser Ca ꭞ, Ser Cys NOS | malignant | IIIa | G3 | 2 (High) | yes | BRCA2+ve |
| 24 | 37 | micropapillary Ser Ca/ Papillary Ser Cys | malignant | IIIc | G1 | I (Low) | yes | no(Nil, father liver Ca --drinker) |
| 25 | 45 | Ser Ca/ Papillary Ser Cys | malignant | IIIc | G1 | I (Low) | yes | n/a |
| 26 | 71 | Ser Ca/ Papillary Ser Cys | malignant | IIIc | G2 | 2 (High) | yes | Nil Ca |
| 27 | 42 | Ser Ca/ Papillary Ser Cys | malignant | IIIc | G2 | 2 (High) | not entered | Family history of Ca |
| 28 | 43 | Ser Ca/ Papillary Ser Cys | malignant | IIIc | G2 | 2 (High) | yes | n/a |
| 30 | 76 | Ser Ca/ Papillary Ser Cys | malignant | IIIc | G3 | 2 (High) | yes | Nil Ca |
| 31 | 54 | Ser Ca/ Papillary Ser Cys | malignant | IIIc | G3 | 2 (High) | yes | BRCA2 +ve |
| 32 | 62 | Ser Ca NOS | malignant | IIIc | G3 | 2 (High) | yes | BRCA1 +ve |
| 33 | 56 | Ser Ca, Ser Cys NOS | malignant | IIIc | G3 | 2 (High) | yes | -ve, Nil Ca |
| 34 | 38 | Ser Ca | malignant | IIIc | G3 | 2 (High) | yes | Nil Ca |
| 35 | 60 | Ser Ca | malignant | IIIc | G3 | 2 (High) | yes | BRCA2 +ve |
| 36 | 59 | Ser Ca | malignant | IV | G2 | 2 (High) | yes | sister with Ca |
| 37 | 61 | Ser Ca | malignant | IV | G2 | 2 (High) | n/a | BRCA2 carrier |

ꭞPrimary site is FT; Ser Ca = Serous Carcinoma; Ser Cys= Serous Cystadenocarcinoma; -ve= negative; +ve= positive; OV = ovaries; FT = Fallopian Tube; Ca = cancer; n/a = not available; G1 = grade 1; G2 = grade 2; G3 = grade 3

**Supplementary Table 1 (B): Clinical information on patients recruited for mRNA study**

| RNA sample # | Patient age at time of diagnosis | Pathology Diagnosis | Tumour origin and type | FIGO stage | Silverberg Grading | WHO (Type) | Patient Genetics and family history | Ascites present at Diagnosis |
| --- | --- | --- | --- | --- | --- | --- | --- | --- |
| 1 | 36 | Ser Cys | OV malignant | Ia | not Graded | 1 (low) | unlikely to be BCRA positive | no |
| 2 | 31 | Ser Cys | OV malignant | Ic | G1 | 1 (low) | Unknown (Schizophrenia; Grandfather lung Ca, grandmother Ca) | no |
| 3 | 33 | Ser Cys, bordeline malignant | OV malignant | Ic | G1 | 1 (low) | Nil Ca | no |
| 4 | 67 | Papillary Ser Cys | OV malignant | IIc | G3 | 2 (High) | unknown | yes |
| 5 | 56 | Ser Cys | OV malignant | IIc | G3 | 2 (High) | BRCA1+ve | yes |
| 6 | 54 | Papillary Ser Cys | OV malignant | IIc | G3 | 2 (High) | unknown (mother Kidney Ca, BrCa) | no |
| 7 | 69 | Ser Cys | OV malignant | IIIa | G2 | 2 (High) | unknown (MC-Sjogren's Syndrome; Uncle Bowel Ca, Uncle leukaemia, Bro Hodgkin's) | no |
| 8 | 56 | Ser Cys | OV malignant | IIIc | G3 | 2 (High) | negative | yes |
| 9 | 83 | Ser Cys | OV malignant | IIIc | G2 | 2 (High) | negative | Yes |
| 10 | 76 | Papillary Ser Cysa | OV malignant | IIIc | G3 | 2 (High) | unknown (Daughter OvCa) | yes |
| 11 | 38 | Ser Cys | OV malignant | IIIc | G3 | 2 (High) | BRCA1+ve | yes |
| 12 | 65 | Papillary Ser Cys | OV malignant | IIIc | G3 | 2 (High) | Nil Ca | yes |
| 13 | 67 | Ser Cys | OV malignant | IIIc | G3 | 2 (High) | Nil Ca | no |
| 14 | 64 | Papillary Ser Cys | OV malignant | IIIc | G3 | 2 (High) | nil Ca | yes |
| 15 | 49 | Ser Cys | OV malignant | IIIc | G3 | 2 (High) | negative | yes |
| 16 | 47 | Papillary Ser Ca | OV malignant | IIIc | G1 | 1 (low) | Unknown (Father liver Ca) | yes |
| 17 | 76 | Ser Cys | OV malignant | IIIc | G3 | 2 (High) | Unknown (Daughter Ovca) | no |
| 18 | 50 | Ser Cys | OV malignant | IV | G3 | 2 (High) | negative | Unknown |
| 19 | 61 | Ser Cys ; Mixed cell adenocarcinoma; Endometrioid carcinoma NOS | OV malignant | IV | G3 | 2 (High) | negative | yes |
| 20 | 57 | Ser Cys | OV malignant | IV | G3 | 2 (High) | negative | yes |
| 21 | 52 | Ser Ca | OV malignant | IV | Undifferentiated/ Anaplastic | 1 (low) | BRCA2+ve | yes |
| 22 | 62 | Ovarian cyst Benign | OV benign | benign | -- |  | unknown | no |
| 23 | 51 | normal ovary | OV benign | benign | -- |  | unknown | no |
| 24 | 62 | Normal Benign | OV benign | benign | -- |  | unknown | no |
| 25 | 37 | Left ovary normal | OV benign | normal | -- |  | unknown | no |
| 26 | 21 | Normal (BRCA1+) | OV benign | normal | -- |  | BRCA1+ve | no |
| 27 | 45 | Normal Ovary (MCLH1 mutation) | OV benign | normal | -- |  | MLH1 carrier | no |
| 28 | 21 | normal ovary | OV benign | normal | -- |  | unknown | no |
| 29 | 55 | Normal (Benign tumour) ovary | OV Benign | benign | -- |  | unknown | no |
| 30 | 63 | Normal Ovary (total hysterectomy) | OV benign | normal | -- |  | unknown | no |

Ser Ca = Serous Carcinoma; Ser Cys= Serous Cystadenocarcinoma; negative = negative for BRAC mutations; +ve= positive; OV = ovaries; Ca = cancer; n/a = not available: Nil Ca= negative for BRAC mutations and no familiar Cancer recorded; G1 = grade 1; G2 = grade 2; G3 = grade

**Supplementary Table 2: Clinical information of ascites samples used for this study**

| Ascites Samples | Fluid or Cell type used  for the study | Pathology Diagnosis | Primary site | FIGO Stage | Silverberg Grade | Age | Time of first recurrence (after completion of first line of chemotherapy) | Time of sample collection (after diagnosis) | Patient  status | Treatment received before  the collection of ascites | Study technique  used |
| --- | --- | --- | --- | --- | --- | --- | --- | --- | --- | --- | --- |
| As59 | Ep & Mesl | Ser Cys NOS | OV | IIc | *G3 | 64 | NA | AD | CN | None | RNA (qRT-PCR) |
| As67 | Mes | adenocarcinoma NOS | MS (not sure, possible OV with distal metastasis to gastrointestinal/ colonic) | Unk | Not graded | 64 | NA | AD | CN | None | RNA (qRT-PCR) |
| As68 | Ep; Fluid | Ser Cys | OV | IV | G3 | 72 | NA | AD | CN | None | RNA (qRT-PCR) & ELISA; |
| As71 | Ep & Mes | Ser Cys NOS | MS (genital tract -Female NEC | Unk | not graded | 62 | NA | AD | CN | None | RNA (qRT-PCR) |
| As77 | Fluid | Carcinosarcoma NOS | MS | IV | G3 | 53 | NA | AD | CN | None | ELISA |
| As80 | Ep & Mesl | Ser Car | FT | Unk | G3 | 53 | NA | AD | CN | None | RNA (qRT-PCR) |
| As81 | Ep & Mes | Serous Papillary Carcinoma | OV | IIIc | G3 | 68 | NA | AD | CN | None | RNA (qRT-PCR) |
| As82 | Ep & Mes; Fuid | Ser Cys | OV | IIIc | *G3 | 48 | NA | AD | CN | None | RNA (qRT-PCR); ELISA |
| As85 | Ep & Mes | Serous cystadenocarcinoma NOS | FT | Unk | G3 | 67 | NA | AD | CN | None | RNA (qRT-PCR) |
| As87 | Fluid | Carcinosarcoma NOS; Ser Cys NOS; Sarcoma NOS | OV | IIIc | G3 | 73 | NA | AD | CN | None | ELISA |
| As95 | Fluid | Ser Cys NOS | OV | Unk | G3 | 71 | NA | AD | CN | None | ELISA |
| As99 | Ep & Mes | Ser Cys NOS | Genital tract | IIIc | G3 | 64 | NA | AD | CN | None | RNA (qRT-PCR) |
| As106 | Fluid | Ser Cys NOS | OV | IIIc | G3 | 41 | NA | AD | CN | None | ELISA |
| As122 | Fluid | Serous cystadenocarcinoma NOS | OV | IIIc | G3 | 60 | NA | AD | CN | None | ELISA |
| As59C | Ep & Mes | Ser Cys NOS | OV | IIc | G3 | 64 | 1 month | 3months 13days | #CR | Carboplatin and Paclitaxel (4 cycles)  Topotecan (1 cycle) | RNA (qRT-PCR) |
| As59D | Fluid | Ser Cys NOS | OV | IIc | G3 | 64 | 1 month | 3 months 20days | #CR | Carboplatin and Paclitaxel (4 cycles)  Topotecan (1 cycle) | ELISA |
| As61 | Mes | Ser Cys NOS | OV | IIIc | G3 | 62 | 1 year 11 months | 2 years 5months | #CR | Carboplatin and paclitaxel (6 cycles),  Carboplatin and paclitaxel (3 cycles)  Cisplatin (4 cycles)  Cyclophosphamide (2 cycles) | RNA (qRT-PCR) |
| As63 | Ep & Mes; Fluid | Adenocarcinoma NOS | OV | IIIc | G3 | 67 | 3years 6 months | 4 years 1 month | #CR | Carboplatin and Paclitaxel (6 cycles),  Tamoxifen (2cycles),  Carboplatin (6 cycles),  cyclophosphamide (2cycles) | RNA (qRT-PCR); Elisa |
| As69 | Ep | Ser Cys NOS | OV | IIIc | G3 | 59 | 3 years 5 months | 4 years 0 months 1 day | #CR | ICON7 Trial [Dexamethasone, Granisetron, Ranitidine, Loratidine, Paclitaxel, Carboplatin, Coloxyl, metoclopramide hydrochloride] (18 cycles); ICON6 Trial [Cycles 1-2:Cediranib, carboplatin, Dexamethasone Cycles 3-6: Aprepitant, Cisplatin, Dexamethasone, Coloxyl, ondansetron hydrochloride] (6 cycles) | RNA (qRT-PCR) |
| As72 | Ep | Papillary Ser Cys | OV | IIIc | *G3 | 62 | 8 months | 2 years 9 months | #CR | Carboplatin and Paclitaxel (6 cycles),  Gemcitabine and Carboplatin  (6 cycles) | RNA (qRT-PCR) |
| As73 | Ep & Mes; Fluid | Ser Cys NOS | OV | IIIc | *G3 | 56 | 6 months | 2 years 9 months | #CR | Doxorubicin Pegylated Liposomal (4 cycles), Carboplatin and Paclitaxel (6 cycles),  AMG-386 182 Trial (9 cycles),  Paclitaxel (6 cycles),  Cyclophosphamide (2 cycles),  Topotecan (2 cycles), Liposomal | RNA (qRT-PCR); ELISA |
| As74 | Ep& Mes | Adenocarcinoma NOS | Genital tract | Unk | Not graded | 85 | 8 months | 1 year 9 months | #CR | Carboplatin (4 cycles),  Cyclophosphamide (10 cycles)  Carboplatin (1 cycle) | RNA (qRT-PCR) |
| As79 | Ep & Mes; Fluid | Papillary Ser Cys | OV | IIIc | G3 | 48 | 2 years 3 months | 2 years 4 months | #CR | Ovar12 (AOCS-PMCC) Trial (8 cycles) | RNA (qRT-PCR); ELISA |
| As84 | Fluid | Mixed cell adenocarcinoma; Ser Cys NOS; Clear cell adenocarcinoma NOS | OV | IIIc | G3 | 58 | 4 years 11 months | 5years | #CR | Paclitaxel and carboplatin (6 cycles),  Carboplatin (6 cycles)  Carboplatin and Gemcitabine Hydrochloride (5 cycles)  Docetaxel and Carboplatin (6 cycles),  Doxorubicin Pegylated Loposomal and carboplatin (8 cycles) | ELISA |
| As91 | Mes | Neoplasm, malignant | OV (Peritoneum-unspecified (first primary) | IIIc | unk | 70 | 11 months | 1 year 1 moth | #CR | Carboplatin and Taxol (6 cycles),  AMG TRINOVA 2 Trial (4 cycles) | RNA (qRT-PCR) |
| As94 | Fluid | Ser Cys NOS | OV | IV | G3 | 71 |  | 2 years 5 months | #CR |  | ELISA |
| As122 | Ep | Ser Cys NOS | OV | IIIc | G3 | 60 | 1 month | 6 months | #CR | Carboplatin and paclitaxel and Bevacizumab (10 cycles) | ELISA, RNA (qRT-PCR) |

^*^G3-Poorly differentiated; NA-Not applicable; #CR - ascites was collected after the patients had undergone recurrence following treatment with the above described cycles of chemotherapy; AD After diagnosis, before treatment; Ser Ca = Serous Carcinoma; Ser Cys = Serous Cystadenocarcinoma; OV = ovary; Ep = epithelial; Mes = mesenchymal; MS = multiple sites.

**Supplementary Table 3: Sequence of primers used in the study**

| Gene symbol | Sequences  (5' - 3') | Accession # | Product size (bp) | Fluorescence Capture (°C) |
| --- | --- | --- | --- | --- |
| 18S | F GTAACCCGTTGAACCCCATT  R CCATCCAATCGGTAGTAGCG | NR_003286.1 | 153 | 78 |
| CD117  (c-Kit) | F GCCCACAATAGATTGGTATTT  R AGCATCTTTACAGCGACAGTC | NM_000222.2 | 570 | 79 |
| EpPCAM | F CGTCAATGCCAGTGTACTTCAGTTG  R TCCAGTAGGTTCTCACTCGCTCAG | NM_002354.2 | 301 | 80-83 |
| MMP-2 | F TTGACGGTAAGGACGGACTC  R ACTTGCAGTACTCCCCATCG | NM_004530.4 | 153 | 80-83 |
| MMP-9 | F TTGACAGCGACAAGAAGTGG  R GCC ATTCACGTCGTCCTTAT | NM_004994.2 | 179 | 83-85 |
| MMP-11 | F AACTGGAGTGTCCTTGCTGT  R AATACCCCTCCCCATTTGAC | NM_005940.5 | 243 | 83 |
| MT1-MMP/  MMP-14 | F GCTCCGAGGGGAGATGTTTG  R CAGCTCCTTAATGTGCTTGGG | NM_004995.2 | 235 | 83 |
| OCT4A | F CTCCTGGAGGGCCAGGAATC  R CCACATCGGCCTGTGTATAT | NM_002701.4 | 381 | 88 |
| PROM1/CD133 | F ATTGGCATCTTCTATGGTTT  R GCCTTGTCCTTGGTAGTGT | NM_006017 | 167 | 78 |
| TIMP-1 | F TGACATCCGGTTCGTCTACA  R GTTTGCAGGGGATGGATAAA | NM_003254.2 | 248 | 85 |
| TIMP-2 | F CCGCAACAGGCGTTTTGCAA  R TCACTTCTCTTGATGCAGGC | NM_003255.4 | 494 | 85 |
| TIMP-3 | F TTCTGCAACTCCGACATCGT  R ATGCAGGCGTAGTGTTTGGA | NM_000362.4 | 452 | 83 |

**Supplementary Table 4: mRNA expression of TIMPs and MMPs with IC_50_ values of paclitaxel (PTX) and cisplatin (CIS) in ovarian cancer cell lines.**

|  |  |  |  |  |  |
| --- | --- | --- | --- | --- | --- |
| Cell line | Origin | TIMPs mRNA expression | MMPs mRNA expression | CIS IC_50_ | PTX IC_50_ |
| JHOS2 | Tumour | TIMP-2 (high)  TIMP-1 (low)  TIMP-3 (low) | MMP-2 (low)  MMP-9 (ND) MMP-14 (low) | 11.86 µmol/ml | 13.62 µmol/ml |
| OVCAR4 | Ascites (after chemotherapy) | TIMP-1(medium)  TIMP-2(medium)  TIMP-3(low) | MMP-2(medium)  MMP-9 (ND)  MMP-14 (low) | 8.73 µmol/ml | 6.36 µmol/ml |
| OVCAR5 | Ascites (before chemotherapy) | TIMP-1(medium)  TIMP-2(medium)  TIMP-3 (ND) | MMP-2 (ND)  MMP-9 (low)  MMP-14 (low) | 15.23 µmol/ml | 2.39 nmol/ml |
| CAOV3 | Tumour | TIMP-1(high)  TIMP-2 (low)  TIMP-3 (low) | MMP-2(ND)  MMP-9 (ND)  MMP-14 (low) | 2.67 µmol/ml | 0.41 µmol/ml |
| HEY | Tumour | TIMP-1(medium)  TIMP-2 (low)  TIMP-3 (very low) | MMP-2 (low)  MMP-9 (high)  MMP-14 (low) | 3.97 µmol/ml | 0.47 nmol/ml |
| SKOV3 | Ascites | TIMP-1(medium)  TIMP-2(medium)  TIMP-3 (low) | MMP-2(medium)  MMP-9 (ND)  MMP-14 (ND) | 13.16 µmol/ml | 0.80 µmol/ml |

ND: Not detected.

**Supplementary Table 5: TCGA data relating to alteration in TIMP-2, -3 and MMP-2, -9, -11 and -14 genes with overall and disease-free survival in patients.**

| **Gene** | **Survival Type** | **Number of Patients** | **# in Altered group** | **# in Unaltered group** | **Median months survival in Altered group (95% CI)** | **Median months survival in Unaltered group (95% CI)** | **p-Value** | **q-Value** |
| --- | --- | --- | --- | --- | --- | --- | --- | --- |
| TIMP-1 | Overall | 1636 | 58 | 1578 | 37.97 (36.20 - 48.72) | 44.91 (43.89 - 47.67) | 0.0684 | 0.274 |
|  | Disease Free | 1172 | 38 | 1134 | 16.13 (14.06 - 34.23) | 18.05 (17.61 - 19.15) | 0.605 | 0.683 |
| TIMP-2 | Overall | 1636 | 72 | 1564 | 49.24 (44.88 - 58.11) | 44.65 (43.43 - 47.54) | 0.325 | 0.585 |
|  | Disease Free | 1172 | 57 | 1115 | 27.76 (21.12 - 39.82) | 17.98 (17.41 - 18.96) | 0.116 | 0.465 |
| TIMP-3 | Overall | 1636 | 20 | 1616 | 44.97 (43.66 - NA) | 44.81 (43.53 - 47.67) | 0.201 | 0.427 |
|  | Disease Free | 1172 | 13 | 1159 | 18.96 (18.00 - NA) | 18.04 (17.51 - 19.15) | 0.567 | 0.755 |
| MMP-2 | Overall | 1636 | 22 | 1614 | 47.57 (43.43 - NA) | 44.81 (43.66 - 47.57) | 0.521 | 0.622 |
|  | Disease Free | 1172 | 17 | 1155 | 10.84 (9.79 - NA) | 18.05 (17.61 - 19.12) | 0.2 | 0.622 |
| MMP-9 | Overall | 1635 | 64 | 1571 | 48.76 (44.97 - 71.74) | 44.65 (43.50 - 47.54) | 0.7 | 0.891 |
|  | Disease Free | 1171 | 46 | 1125 | 25.00 (18.96 - 31.56) | 17.97 (17.28 - 18.96) | 0.246 | 0.891 |
| MMP-11 | Overall | 1636 | 27 | 1609 | 43.40 (39.39 - NA) | 44.84 (43.79 - 47.57) | 0.569 | 0.925 |
|  | Disease Free | 1172 | 19 | 1153 | 22.77 (17.71 - NA) | 18.02 (17.51 - 18.97) | 0.925 | 0.925 |
| MMP-14 | Overall | 1636 | 55 | 1581 | 34.79 (33.44 - 36.60) | 45.30 (44.29 - 48.06) | 5.41E-04 | 2.16E-03 |
|  | Disease Free | 1172 | 37 | 1135 | 13.08 (12.32 - 18.04) | 18.18 (17.77 - 19.55) | 4.56E-03 | 9.11E-03 |
